# High ETV6 Levels Support Aggressive B Lymphoma Cell Survival and Predict Poor Outcome in Diffuse Large B-Cell Lymphoma Patients

**DOI:** 10.3390/cancers14020338

**Published:** 2022-01-11

**Authors:** Dario Marino, Marco Pizzi, Iuliia Kotova, Ronny Schmidt, Christoph Schröder, Vincenza Guzzardo, Ilaria Talli, Edoardo Peroni, Silvia Finotto, Greta Scapinello, Angelo Paolo Dei Tos, Francesco Piazza, Livio Trentin, Vittorina Zagonel, Erich Piovan

**Affiliations:** 1 Medical Oncology 1, Veneto Institute of Oncology, IOV-IRCCS, 35128 Padova, Italy; dario.marino@iov.veneto.it (D.M.); silvia.finotto@iov.veneto.it (S.F.); vittorina.zagonel@iov.veneto.it (V.Z.); 2 Surgical Pathology & Cytopathology Unit, Department of Medicine—DIMED, University of Padova, 35128 Padova, Italy; marco.pizzi.1@unipd.it (M.P.); vincenza.guzzardo@unipd.it (V.G.); angelo.deitos@unipd.it (A.P.D.T.); 3 Sciomics GmbH, 69151 Neckargemünd, Germany; iuliia.kotova@sciomics.de (I.K.); ronny.schmidt@sciomics.de (R.S.); schroeder@sciomics.de (C.S.); 4 Department of Surgery, Oncology and Gastroenterology, University of Padova, 35128 Padova, Italy; ilaria.talli@studenti.unipd.it; 5 Immunology and Molecular Oncology Unit, Veneto Institute of Oncology, IOV-IRCCS, 35128 Padova, Italy; edoardo.peroni@iov.veneto.it; 6 Hematology Unit, Department of Medicine—DIMED, University of Padova, 35128 Padova, Italy; greta.scapinello@gmail.com (G.S.); francesco.piazza@unipd.it (F.P.); livio.trentin@unipd.it (L.T.)

**Keywords:** B-cell lymphoma, apoptosis, forward phase protein arrays, diffuse large B-cell lymphoma

## Abstract

**Simple Summary:**

B-cell lymphomas are tumors that arise from the proliferation of altered B-cells in lymphoid organs. Diffuse large B-cell lymphoma (DLBCL) is the most common form of lymphoma and is very heterogeneous from a molecular, genetic and clinical point of view. To identify novel therapeutic targets, we compared protein expression levels of selected molecules from biopsies of DLBCL patients with different clinical outcomes. We found that two proteins were particularly important for predicting patient survival. The evaluation of these two proteins improves the capacity to discriminate which patients show prolonged survival or succumb of disease. Furthermore, expression levels of one of these proteins predicts sensitivity to a specific death-inducing drug, suggesting the possibility of personalized treatment.

**Abstract:**

The identification of prognostic factors for aggressive B-cell lymphomas still represents an unmet clinical need. We used forward phase protein arrays (FFPA) to identify proteins associated with overall survival (OS) from diagnostic formalin-fixed paraffin-embedded material of diffuse large B-cell lymphoma (DLBCL) patients (*n* = 47). Univariate Cox regression analysis identified numerous proteins, including immune check-point molecules (PDCD1, PDCD2 and PD1L2) and BCL2 to be significantly associated with OS. However, only ETV6 and PIM2 proteins persisted following multivariate Cox analysis. Independent validation studies by immunohistochemistry and analysis of public gene expression profiles of DLBCL confirmed a prognostic role for high ETV6 and ETV6/PIM2 ratios in DLBCL. *ETV6* is a recurrently mutated/deleted gene in DLBCL for which its function in this disease entity is currently unknown. We find that ETV6 is upregulated during oncogenic transformation of germinal center B-cells and that it regulates DLBCL survival, as its acute loss results in marked apoptosis. Fluctuations in survivin (BIRC5) expression levels were associated with this phenomenon. Furthermore, an inverse correlation between ETV6 and BIRC5 expression levels was found and correlated with a response to the BIRC5 inhibitor, YM155. In conclusion, we present evidence for an oncogenic function of ETV6 in DLBCL.

## 1. Introduction

Diffuse large B-cell lymphoma (DLBCL) is the most common B-cell neoplasm throughout the world, representing 30–35% of all B-cell lymphomas. DLBCL is a biologically aggressive and heterogeneous disease, with a cure rate of approximately 60% [1,2,3]. DLBCLs comprise different molecular entities including DLBCL not otherwise specified (DLBCL NOS) and primary DLBCL of the central nervous system (DLBCL CNS) [4]. Primary mediastinal (thymic) large B-cell lymphoma (PMBL) is a closely related entity, which the World Health Organization (WHO) separates from other large B-cell lymphomas on the basis of its peculiar clinical, immunophenotypic and molecular features [5]. DLBCL NOS has been classified according to gene expression profiling (GEP) into two distinct molecular cell-of origin (COO) subtypes: germinal center B-cell (GCB) and activated B-cell (ABC). About 10–15% of cases cannot be included in either of these subtypes and remain unclassified (UNCL) [6,7,8]. The COO subtypes are associated with very different prognoses (worst for ABC-subtype). The microarray-based GEP technique used for COO determination was originally described using RNA extracted from frozen tissue. The limited access to frozen tissues and the long turnover time have, however, limited its application, and other techniques using formalin-fixed paraffin-embedded (FFPE) tissue have been developed during the last decade. These include immunohistochemistry (IHC)-based classifications [9,10], quantitative reverse transcription PCR (qRT-PCR) [11,12,13,14] and digital GEP (Lymph2Cx) assays [15]. These methods, in particular, IHC-based classifications, have shown rather mixed results as prognostic tools [16]. For clinical DLBCL risk stratification, additional quantitative methods are, thus, required to improve outcome and identify personalized therapeutic targets. One such approach relies on protein levels rather than mRNA transcripts from FFPE samples by using “state-of-the-art” custom forward phase protein arrays (FPPA) [17]. Antibody microarrays are a new tool for the evaluation of protein abundance in a parallel and highly multiplex manner [18]. Through FFPA technology our aim was to identify proteins implicated in DLBCL pathophysiology, correlating with clinical aggressiveness and possibly representing novel therapeutic targets.

## 2. Materials and Methods

### 2.1. Primary Tumor Specimens

We initially evaluated 47 patients with newly diagnosed DLBCL at the Veneto Institute of Oncology (IOV) from July 2011 to November 2016 with sufficient tissue for proteomics analysis. For validation studies, we evaluated another 49 DLBCL patients. Written informed consent was obtained from all subjects, and the analysis was approved by local ethics committee. All experiments conformed to the principles set out in the WMA Declaration of Helsinki. Histological diagnosis was reviewed according to the 2016 WHO classification of lymphoid neoplasms [5]. We also included three cases of primary DLBCL CNS.

### 2.2. Forward Phase Protein Arrays

Antibody microarrays were produced and used according to protocols and strict quality control procedures, as reported earlier [17,19]. For the analysis, a set of 82 target proteins that represent the translational products of transcripts implicated in DLBCL pathobiology, such as those defining COO, oncogenes and drug targetable targets [11,13,14,20]. Antibodies targeting 82 unique proteins were purchased from different sources or provided by collaborating partners. A complete list of binders is provided in the Supplementary Materials (Supplementary Table S1). In this pilot study, FFPE tissue material from 47 patients with DLBCL (including three cases of primary DLBCL CNS) and 3 reactive lymph nodes (LNF) were obtained. The samples were labelled at an adjusted protein concentration with scioDye1 and scioDye2 and washed and hybridised to antibody microarrays in a dual-colour approach using a reference-based design (Sciomics). For competitive dual-colour incubations, a reference sample was produced by pooling the same amount of all protein samples. The same reference sample was used throughout the analysis. After 3 h incubation, slides were washed and subsequently dried with nitrogen before being scanned using a Powerscanner (Tecan, Austria). Differences in protein abundance between samples or sample groups were represented as log-fold changes calculated for the base 2.

### 2.3. Histological Evaluation and Immunohistochemical Analysis

All cases (discovery and validation cohort) were retrieved from the archives of the Surgical Pathology & Cytopathology Unit of Padua University Hospital (Padua, Italy). Each case was re-evaluated and assigned to a COO subtype, according to the Hans algorithm (immunostain for CD10, Bcl6 and MUM1). Representative histological samples were selected for further phenotypic characterization (i.e., assessment of PIM2 and ETV6 expression). In detail, IHC analysis was performed on 4 μm-thick FFPE sections with the Bond Polymer Refine Detection kit in an automated immunostainer (BOND-MAX system; Leica Biosystems—Newcastle upon Tyne, UK), as previously described [21]. Immunostains were performed on whole tissue sections. Where necessary, tissue microarrays were prepared as described previously [22]. TMA blocks were prepared using the Galileo TMA CK3500 (Integrated System Engineering, Milan, Italy; Padova University Hospital) arrayers. Appropriate positive and negative controls were also included. Phenotypic studies on benign palatine tonsils with reactive lymphoid hyperplasia (*n* = 5) were run in parallel to assess ETV6 and PIM2 expression in normal B-cell subsets. We defined the following four-tiered scoring system for ETV6 and PIM2 expression: (i) score 0: no staining or weak positivity in <20% of tumor cells; (ii) score 1+: weak positivity in ≥20% of tumor cells; (iii) score 2+: moderate positivity in ≥20% of tumor cells; (iv) score 3+: strong positivity in ≥20% of tumor cells. The scoring system was based on the nuclear expression of both markers, and intensity scores were defined by comparison with positive controls (i.e., squamous epithelium of palatine tonsils). Specifically, strong (score 3+) positivity was attributed to DLBCL cases with protein expression comparable to that of squamous epithelia of palatine tonsils, moderate (score 2+) positivity to cases with protein expression slightly fainter than controls and weak (score 1+) positivity to cases with barely detectable protein expression. To allow comparison among groups, DLBCL cases were lumped together based on ETV6 and PIM2 positivity scores as follows: (i) low expressing cases (immunohistochemical score 0 and 1+); (ii) high expressing cases (immunohistochemical score 2+ and 3+).

The following primary antibodies were used: anti-CD10 (clone 56C6, Menarini Diagnostics, Florence, Italy); anti-Bcl6 (clone LN22, Leica Biosystems, Milan, Italy); anti-MUM1 (clone MUM1p, Dako, Glostrup, Denmark); anti-PIM2 (clone D-8, Santa Cruz Biotechnology, Dallas, TX, USA); and anti-ETV6 (HPA000264, Sigma-Aldrich, St. Louis, MO, USA).

### 2.4. Bioinformatical Software and Analyses

Gene Set Enrichment Analysis (GSEA) using WEB-based GEne SeT AnaLysis Toolkit (WebGestalt [23]) with Wikipedia cancer pathway as enrichment categories was performed on all proteins significantly associated with OS (*n* = 41). Network Topology-based analysis (NTA; another module present in WebGestalt) for 41 prognostic proteins using the TCGA RNA Seq data for DLBCL samples (*n* = 48) as functional database was used to identify relevant connecting sub-networks. GeneMANIA [24] is a web interface that uses large sets of functional association data to identify single genes related to a set of input genes. Association data include protein and genetic interaction pathways, co-expression, co-localization and protein domain homology. GeneMANIA was used to contruct the ETV6, PIM2 and ETV6-PIM2 biological network. The list of identified co-expressed/interacting genes of ETV6 was used to run an over-representation analysis (ORA) using Reactome pathways as functional database to identify if they associate into certain pathways.

### 2.5. Gene Expression Data

Gene expression data of DLBCL patients analyzed with HGU133+2.0 Affymetrix GeneChip arrays (*n* = 223) was obtained from Gene Expression Omnibus (GSE873371) [25]. PIM2 and ETV6 expression levels were extracted and used to generate Kaplan–Meier survival plots. Gene expression changes in the B-cell lymphoma cohort [26] were obtained from cBioPortal (a tool developed by the Computational Biology Center at Sloan Kettering) [27,28], and the results were presented as OncoPrint format data and used to generate Kaplan–Meier survival plots. Gene expression data for normal and malignant B-cells (DLBCL) were extracted from GSE56315 [29]. Genetic alterations and gene expression levels associated with selected genes in B-lymphoma cell lines [30] were also obtained by using cBioPortal.

### 2.6. Western Blotting

For Western blotting, protein samples were separated on 4–12% gradient Tris-Glycine or 12% Tris-Glycine SDS-PAGE Gels (Invitrogen, Thermo Fisher Scientific, Waltham, MA, USA) and transferred to PVDF membrane (Millipore, Billerica, MA, USA). Antibodies against tubulin (TU-02; Santa Cruz Biotechnology), PIM2 (MAB4355, R&D Systems, Minneapolis, MN, USA or HPA000285, Sigma-Aldrich), ETV6 (HPA000264, Sigma-Aldrich), β-actin (#4970; Cell Signaling Technologies, Danvers, MA, USA), XIAP (#14334; Cell Signaling Technologies), cleaved PARP-1 (#5625; Cell Signaling Technologies) and survivin/BIRC5 (#2808; Cell Signaling Technologies) were used. The BioRad ChemiDoc XRS Imager was used to capture signals from blots. We quantified each protein band using ImageJ software (National Institutes of Health, Bethesda, MD, USA) and normalized each target protein after background subtraction to its loading control (β-actin or tubulin).

### 2.7. Lentiviral Constructs and Viral Production

Human *ETV6* knock-down (KD) was performed using pLKO.1-sh*ETV6*-puro constructs (#1: TRCN0000003854; #2: TRCN0000003856; Sigma-Aldrich). pLKO.1-control (SHC007, SHC002; Sigma-Aldrich) were used as controls (CTRL). For viral production, appropriate expression plasmids were transfected in HEK293T cells using JetPEI transfection reagent (Polyplus, Illkirch, France) together with packaging plasmids. The viral supernatant was collected 48 h after transfection, filtered and used to infect target cells. All infections were performed by spinoculation. After infection, DLBCL cell lines were selected for 5–7 days in puromycin before functional assays

### 2.8. Cell Viability Assays and Flow Cytometry

Cell viability in DLBCL cell lines treated with different concentrations of YM155 (Selleck Chemicals LLC, Houston, TX, USA) was analyzed after 48 h via the bioluminescent method Vialight plus (Lonza, Basel, Switzerland). This assay allows bioluminescent detection of cellular ATP as a measure of viability. We analyzed apoptosis 48 h post completion of puromycin selection by flow cytometry (FACS) after staining with Annexin-V-FLUOS Staining Kit (Roche, Basel, Switzerland) and SYTOX Red dead cell stain (Thermo Fischer Scientific). Apoptosis was defined as the sum of the percentage of Annexin V+ and Annexin V+/SYTOX Red+ cells. The samples were collected on a FACS Calibur (BD Biosciences, Milan, Italy) using Cell Quest software (BD Biosciences) and analysed with FlowJo™ Software (FlowJo LLC, Ashland, OR, USA).

### 2.9. Statistical Analyses

We performed statistical analysis by Student’s t-test and Mann–Whitney U test where appropriate. A non-parametric test (Chi-square test) was used to compare qualitative data, including clinical variables presented in Table 1. All statistical tests were two sided and unpaired and *p* < 0.05 was considered statistically significant. Linear regression analyses and Pearson’s correlation coefficients were conducted for calculating correlations (GraphPad Prism Software, San Diego, CA, USA). Statistical analysis of protein expression data to identify clinically relevant prognostic proteins was also performed using the Cox regression analysis (MedCalc Software bv, Ostend, Belgium; https://www.medcalc.org; accessed on 9 November 2018). The Kaplan–Meier method was used to estimate the distributions of OS. OS was considered as the time from diagnosis to date of death or last follow-up. The log-rank test or Gehan–Breslow–Wilcoxon test was used to compare survival distributions.

Methods concerning cell lines, array production, sample labeling and hybridization, array data analysis and statistical testing, Nanostring Assay for COO, Western blotting and quantitative real time RT-PCR are detailed in Supplementary Materials and Methods.

## 3. Results

### 3.1. Antibody Arrays Identify Two Distinct Protein Signatures in Profiled DLBCLs

**Figure 1:**
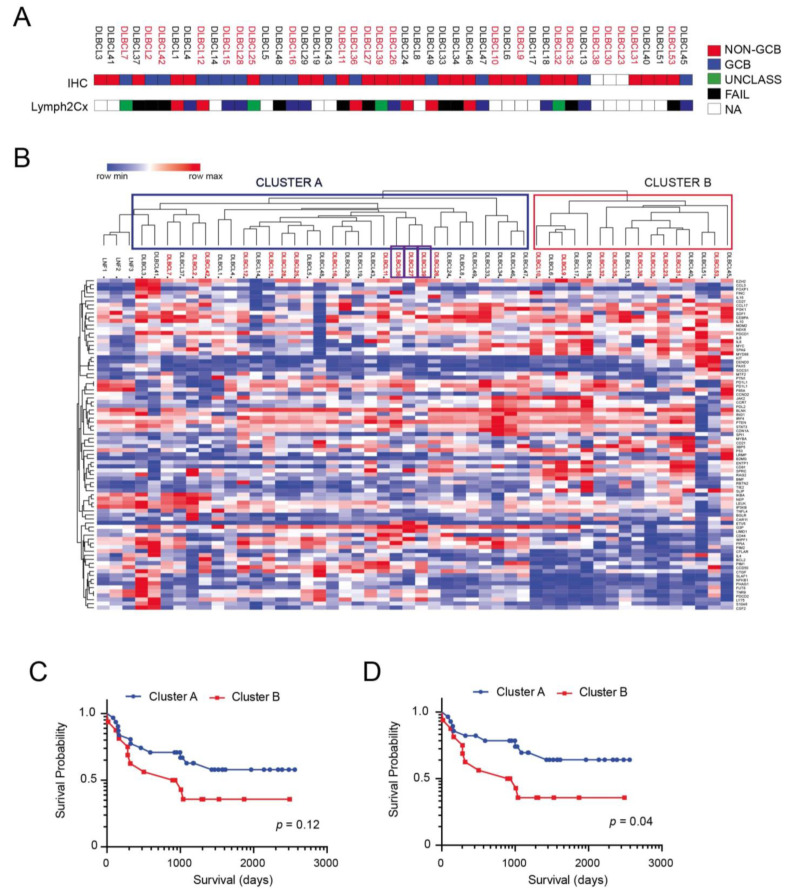
Performance of FFPA in stratifying DLBCL patient samples. (**A**) Cell-of-origin (COO) subtyping from the Hans IHC algorithm and Lymph2Cx assay for the cohort of patient samples (*n* = 47). (**B**) Unsupervised hierarchical Clustering of DLBCL samples based on differentially abundant proteins. Samples labeled in red correspond to patients which had an adverse event (death). Primary DLBCL CNS cases are identified by purple boxes. (**C**) Kaplan–Meier curves of OS in the two clusters (Cluster A and B) identified by using FFPA analysis. (**D**) Kaplan–Meier curves of OS in the two clusters (Cluster A and B) identified by using FFPA analysis following the exclusion of primary DLBCL CNS cases.

The clinicopathological features of the 47 DLBCL cases of the discovery cohort are summarized in Table 1. The clinical characteristics between patients with complete remission (CR) after first line chemotherapy and those with partial response (PR) or progressive disease (PD) according to Lugano Criteria for response evaluation in Lymphoma [31] demonstrated clear differences in Eastern Cooperative Oncology Group Performance Status (ECOG-PS) and Comprehensive Geriatric Assessment (CGA) scores. Specifically, our cohort consisted of 24 males and 23 females. The median age at diagnosis was 69 years (20–83). Of these, 20 patients (42%) had low (score 0–1), 20 (42%) had intermediate (2–3) and 7 (15%) had high (4–5) IPI risk scores. IHC was informative in 44/47 (93%) patient samples. Twenty-seven out of forty-four (61%) were assigned to non-GCB subtype, while the remaining 17/44 (39%) were classified as GCB subtype according to Hans algorithm (Figure 1A). Out of thirty-three cases investigated with the Nanostring Lymph2Cx assay for COO determination, 21 were successfully characterized while 12 cases failed to pass the analysis due to insufficient RNA quality (Figure 1A). Ten cases (48%) were classified as GCB, seven cases (33%) were classified as ABC, and four cases (19%) were classified as UNCL (Figure 1A). Furthermore, 9 patients (19%) showed ECOG-PS ≥ 2, 28 (59%) showed stage III or IV disease, 5 (10%) showed >1 extra nodal site, 19 (40%) showed elevated LDH and 17 (36%) showed bulky disease (>6 cm).

Most patients received the combination rituximab plus cyclophosphamide, doxorubicin, vincristine and prednisone (R-CHOP) or CHOP-like treatments, and one patient received hyper-fractionated (Hyper) CVAD because of CNS involvement at diagnosis. Thirty-three patients obtained CR after the end of first line chemotherapy; however, 10 patients progressed during follow-up. On the other hand, 14 patients presented with PR or PD after the end of first line chemotherapy. The follow-up period ranged from 16 days to 2491 days (6.8 years) with the median being 1009 days (2.76 years). During this follow-up period, 13 patients succumbed to disease.

To gain insight on factors associated with clinical aggressiveness and which could represent optimal therapeutic targets, we selected a group of 82 unique proteins corresponding to transcripts previously implicated in DLBCL COO classification and pathophysiology [11,13,14,20] for which antibodies of suitable specificity were available at the moment of array production (2017; see Table S1).

We studied the expression levels of the listed proteins in all 47 DLBCL patients for which survival data were available and in three reactive lymph nodes (LNF). We performed hierarchical clustering analysis of samples based on differentially expressed proteins (*n* = 82), which identified two main clusters (Figure 1B). One of the clusters (Cluster A; *n* = 31) is highly related to reactive B-cell lympho-proliferations (LNF1-3), while the other (Cluster B; *n* = 16) is not related to this protein signature. Interestingly, primary DLBCL CNS cases, although showing some differences in protein expression compared to DLBCL NOS (Figure S1), did not form a separate cluster but were included within Cluster A. The Kaplan–Meier survival analysis disclosed that patients belonging to Cluster A had a worse clinical outcome, although not statistically significant (Figure 1C; *p* = 0.12), possibly for the low number of samples analyzed. Peculiarly, this difference became significant when DLBCL CNS cases were excluded from the analysis and only DLBCL NOS were considered (Figure 1D; *p* = 0.04).

### 3.2. ETV6 and PIM2 Represent Candidate Proteins Predicting Survival in Aggressive DLBCLs Cases

To identify proteins associated with clinical outcome in our cohort of DLBCLs, we followed the workflow outlined in Figure S2A. We first performed a univariate Cox regression analysis of protein expression data coupled with OS as the dependent variable. This analysis identified 41 unique proteins (50%; 41/82) to be significantly associated with OS (Figure S2B and Table 2).

**Figure 2:**
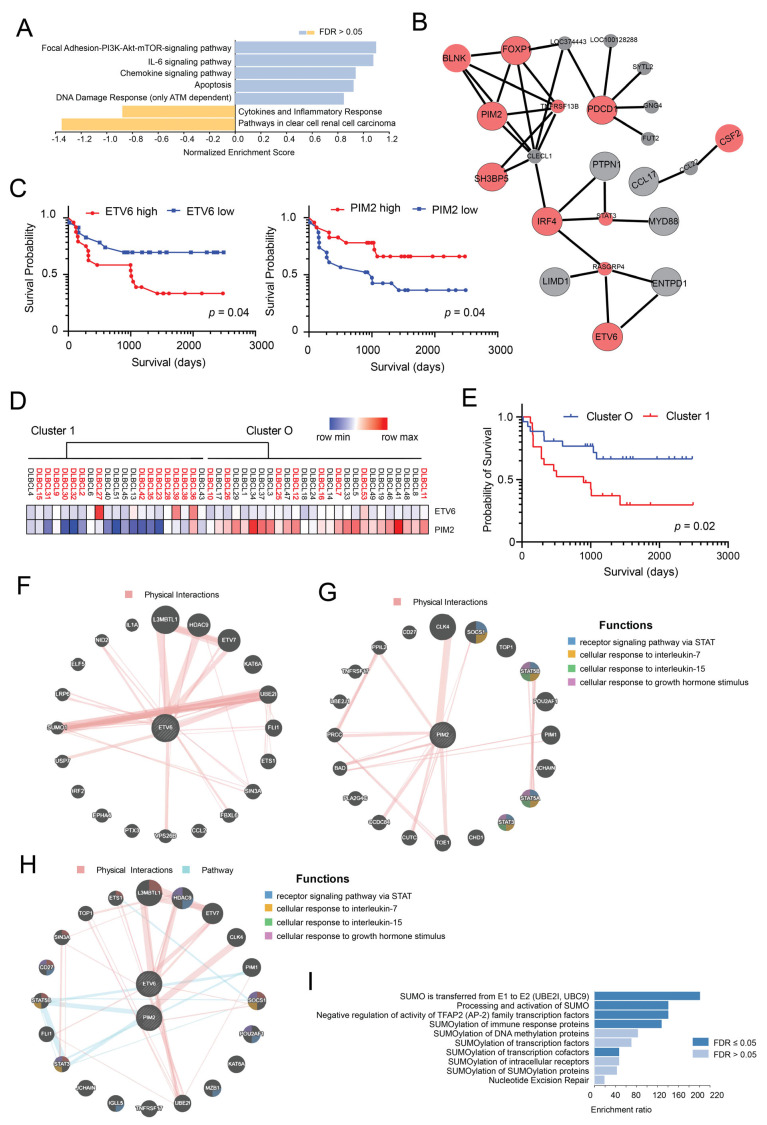
Identification of proteins associated with survival through univariate and multivariate Cox regression analysis. (**A**) The list of proteins identified as associated with survival through univariate Cox regression analysis (*n* = 41) was used for Gene Set Enrichment Analysis (GSEA) using WEB-based GEne SeT AnaLysis Toolkit (WebGestalt) with Wikipedia cancer pathway as enrichment categories. The enriched categories are shown. (**B**) Network topology-based analysis for the same list of proteins was run using TCGA RNA Seq data for DLBCL samples as the functional database. The identified network was found to be enriched for numerous Gene Ontology (GO) categories, including immune system development (proteins included in this subnetwork are shown). (**C**) Kaplan–Meier curves of OS in patients (*n* = 47) subdivided into two groups on the basis of ETV6 protein expression levels (left panel) or PIM2 protein expression levels (right panel). High: >median value; low: ≤median value. (**D**) Unsupervised hierarchical clustering of DLBCL samples using ETV6 and PIM2 proteins. (**E**) Kaplan–Meier curves of OS in the two clusters (Cluster 1 and 0) identified using ETV6 and PIM2 protein levels. (**F**) Biological interaction network of ETV6 showing physical interactions obtained using GeneMANIA. (**G**) Biological interaction network of PIM2 showing physical interactions and associated functional pathways obtained using GeneMANIA. (**H**) Biological interaction network of ETV6 and PIM2 showing physical interactions and associated functional pathways obtained using GeneMANIA. (**I**) Over representation analysis (ORA) of ETV6 interacting proteins using Reactome pathways as functional database.

GSEA using WEB-based GEne SeT AnaLysis Toolkit (WebGestalt) of these unique proteins disclosed enrichment for pathways (Figure 2A), including the following: Focal Adhesion-PI3K-Akt-mTOR-signaling pathway, IL-6 signaling pathway, Chemokine signaling pathway, Apoptosis, DNA Damage Response (only ATM dependent) and Cytokines and Inflammatory Response. Network Topology-based analysis (NTA) for the 41 prognostic proteins using TCGA RNA Seq data for DLBCL samples as functional database identified a subnetwork. This network was enriched for numerous Gene Ontology (GO) categories, including immune system development (sub-network of proteins in this category are shown in Figure 2B), immune system process and cytokine-mediated signaling pathway. Stepwise multivariate survival analysis performed by the Cox proportional hazards model of the 41 proteins from the previous step selected two proteins (ETV6 and PIM2) as independent prognostic factors (Table 3). ETV6 was associated with worse prognosis (*p* = 0.028), while PIM2 was associated with better outcomes (*p* = 0.034). Interestingly, both genes have been shown to be upregulated in the ABC-type DLBCL subgroup [32].

Kaplan–Meier survival curves confirmed the prognostic relevance of ETV6 and PIM2 protein expression in our cohort of DLBCL samples (Figure 2C). Furthermore, unsupervised hierarchical clustering of DLBCL samples using these two proteins (ETV6, PIM2) efficiently subdivided the DLBCL samples in two defined clusters associated with different survival (Figure 2D,E). Interestingly, Cluster 1 (Figure 2E) contained most patients previously falling in Cluster B (Figure 1C). Western blot analysis and IHC staining of selected samples for which sufficient material was available provided rather concordant results to those observed using antibody arrays (Figure S3A–E). Coherently, lower levels of PIM2 and higher levels of ETV6 in patient samples were associated with poor survival (Figure S3F,G). Given these results, we constructed an interaction network for ETV6 and PIM2 to identify potential interactions between these two proteins and other cancer-associated proteins (Figure 2F,G) or potential interactions between them (Figure 2H). We then used the list of genes co-expressed/interacting with ETV6 to run an ORA analysis to identify enriched gene sets. We found enrichment for several gene sets related to SUMOylation of transcription factors, suggesting a role for ETV6 in regulating SUMOylation (Figure 2I) in DLBCLs.

### 3.3. Generation of a Model Based on the Expression of PIM2 and ETV6 for Predicting Survival in DLBCL

**Figure 3:**
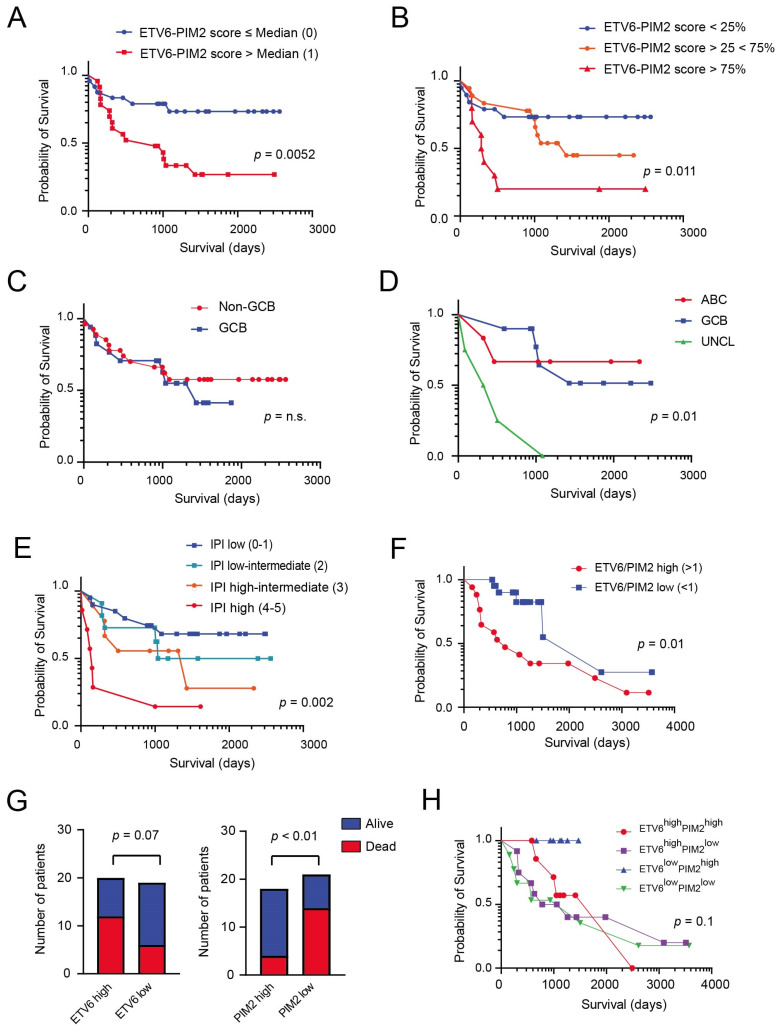
Kaplan–Meier curves of OS in DLBCL patients treated with chemotherapy and stratified according to various putative prognostic markers. (**A**) Kaplan–Meier curves of OS in two groups identified using the PIM2-ETV6 prognostic score (Group 0: score ≤ median; Group 1: score > median). (**B**) Kaplan–Meier curves of OS in the three groups identified using the tertiles of PIM2-ETV6 prognostic score (<25%, 25–75%, >75% of score). (**C**) Kaplan–Meier curves of OS in the same patients subdivided into GCB and non-GCB subgroups using the Hans immune-histochemical algorithm, which uses three markers (CD10, BCL6 and MUM1) to separate GCB DLBCL from non-GCB DLBCL. (**D**) Kaplan–Meier curves of OS in the same cohort of patients subdivided into COO subgroups (ABC, GCB, UNCL) using the Lymph2Cx assay. (**E**) Kaplan–Meier curves of OS in the same patients subdivided into 4 groups using the IPI clinical score. (**F**) IHC validation of ETV6 and PIM2 expression in an independent DLBCL patient cohort (*n* = 39). Kaplan–Meier curves of OS in this cohort, dividing the patients into two groups in relations to the ETV6/PIM2 ratio (high: ratio > 1; low < 1). (**G**) Bar graph showing the distribution of patients’ outcome (alive versus dead) in relation to ETV6 or PIM2 expression. High: staining intensity > 1; low: staining intensity ≤ 1. The Chi square test was used to determine significance. (**H**) Kaplan–Meier curves of OS in the four groups identified using the PIM2 (low versus high) and ETV6 (low versus high) IHC expression levels (cohort *n* = 39).

Given these promising results, we constructed a two-protein prognostic model based on the relative contribution of PIM2 and ETV6 obtained using the multivariate analysis, and described in the following equation: PIM2-ETV6 mortality score = (−0.2927 × PIM2 + 0.6489 × ETV6). The median score in this discovery set was −0.148326. We ranked the patients according to their PIM2-ETV6 scores and divided them based on the median score into two groups: score ≤ median (24 patients) and score > median (23 patients). There was a statistically significant difference in OS between these risk groups (*p* = 0.0052; Figure 3A). Furthermore, by subdividing patients in tertiles of PIM2-ETV6 mortality score (<25%, 25–75%, >75% of score), we were able to stratify patients with distinct outcomes (*p* = 0.011; Figure 3B). We compared the performance of our PIM2-ETV6 score with the widely used methods for COO identification: the Hans IHC algorithm, which uses three markers (CD10, BCL6 and MUM1) and the Lymph2Cx assay. As shown in Figure 3C, the IHC algorithm used to identify COO was not of prognostic significance in our cohort. On the other hand, COO identification using Lymph2Cx was of prognostic importance, although it was the unclassified (UNCL) group that showed the worst prognosis (Figure 3D; *p* = 0.01). As expected, there was a statistically significant difference in OS between the risk groups identified by IPI score (Figure 3E; *p* = 0.002). We next examined whether the prognostic significance of the 2-protein model, based on the ETV6-PIM2 score was independent of the IPI score. A multivariate Cox regression analysis that included IPI scores and ETV6-PIM2 scores with OS as the dependent variable was performed. This analysis disclosed that both the IPI and ETV6-PIM2 scores were independent predictors of OS (Table 4).

We subsequently proceeded to validate our main findings in an independent cohort of DLBCL patients (*n* = 49 of which 39 resulted informative) using IHC (Figure 3F) given the low number of proteins for evaluation (*n* = 2) and its possible subsequent routine clinical application. We found that lymphoma patients with high ETV6/PIM2 ratios (>1) had a worse outcome compared to patients with low ETV6/PIM2 ratios (<1; *p* = 0.01; Figure 3F). This ratio was more effective in predicting survival compared to single proteins (ETV6 (staining intensity > 1; Chi-square, two sided *p* = 0.07) and PIM2 (staining intensity < 2; Chi-square, two sided *p* < 0.01)) (Figure 3G,H).

### 3.4. External Validation and Prognostic Significance of ETV6 Overexpression in Aggressive B-Cell Lymphomas

**Figure 4:**
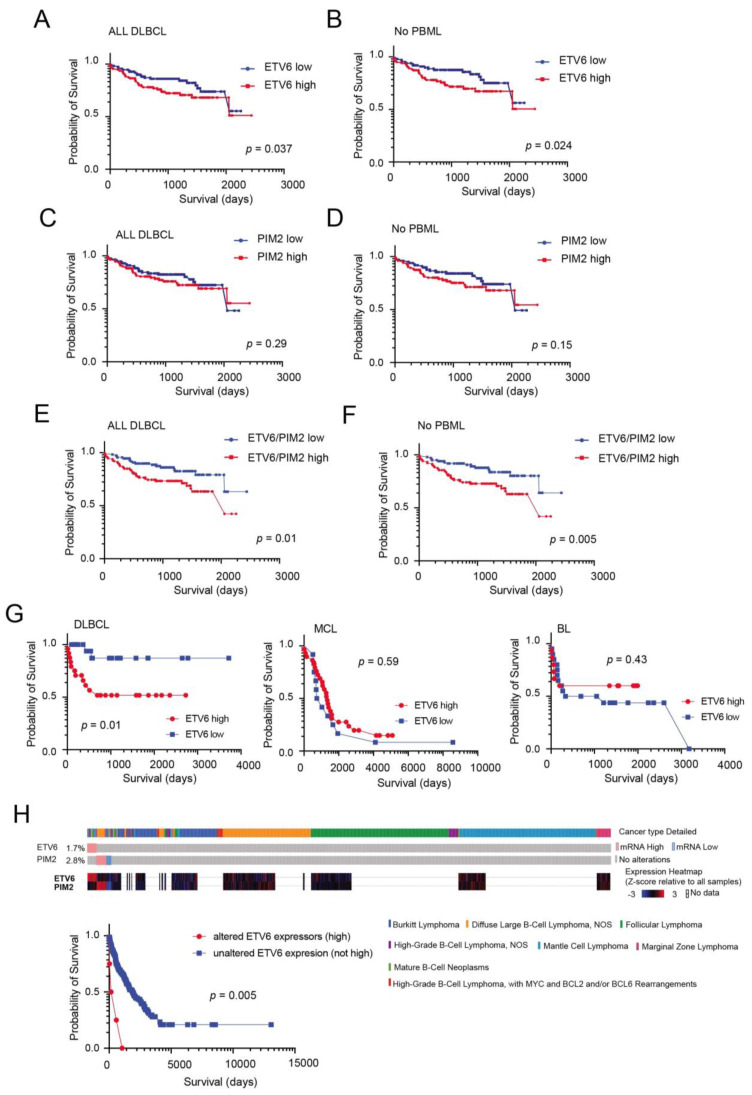
High ETV6 or ETV6/PIM2 levels predict poor survival in B-cell lymphomas. Kaplan–Meier survival curves of the entire series of 223 DLBCL patients [25], analysed as a group (**A**) or excluding PBML cases (**B**). ETV6 (probe 235056_at) high cases (>median expression) or ETV6 low cases (<median expression). Kaplan–Meier survival curves of the entire series of 223 DLBCL patients [25], analysed as a group (**C**) or excluding PBML cases (**D**). PIM2 (probe 204269_at) high cases (>median expression) or PIM2 low cases (<median expression). Kaplan–Meier survival curves of the entire series of 223 DLBCL patients [25], analysed as a group (**E**) or excluding PBML cases (**F**). High ETV6/PIM2 ratio (>median level) or low ETV6/PIM2 ratio cases (<median expression). (**G**) Kaplan–Meier survival curves of a series [26] of DLBCL patients (left panel), mantle cell lymphoma (MCL) patients (middle panel) or Burkitt lymphoma (BL) patients (right panel). ETV6 high cases (>median expression) or ETV6 low cases (<median expression). (**H**) OncoPrint in cBioPortal identified ETV6 high expressors (>2 mean expression Z score) across the B-cell lymphoma subtypes [26] (top panels), and these cases are associated with poor survival (bottom panel).

In order to further explore the relevance of our proteomics findings, we took advantage of published gene expression data on an extensive cohort of well characterized DLBCL patients (*n* = 223) [25]. This cohort includes different molecular DLBCL subtypes: GCB, ABC, UNCL and PMBL cases. Dichotomizing ETV6 transcript expression levels (above and below median expression levels) in order to identify ETV6 high and low cases, we found that ETV6 levels influenced prognosis of these patients (Figure 4A). This phenomenon was even more pronounced when PMBL cases were excluded from the analysis (Figure 4B). On the other hand, PIM2 transcript levels did not impact the survival in this cohort even when PMBL cases where excluded from the analysis (Figure 4C,D). Furthermore, we found that the ETV6/PIM2 ratio (above versus below median score) was highly effective in predicting survival (Figure 4E,F), much as previously described for our IHC validation studies. Similar results were obtained using an independent probe set for ETV6 (Figure S4). We then took advantage of a recently published study [26] where common subtypes of B-cell lymphomas (including DLBCL) have been comprehensively interrogated at the genomic and transcriptional level to determine whether ETV6 levels may also hold prognostic relevance in these different disease entities. Although only a limited number of samples had complete transcriptional, genomic and clinical data, we found that dichotomizing ETV6 levels (above/below median expression) was of prognostic importance only in DLBCL patients but not in BL or MCL patients (Figure 4G). Interestingly, however, patients with very high levels of ETV6 (Z score expression > 2) were associated with exceedingly low survival (independently from disease classification; Figure 4H).

### 3.5. ETV6 Expression in Normal and Malignant B-Cells

**Figure 5:**
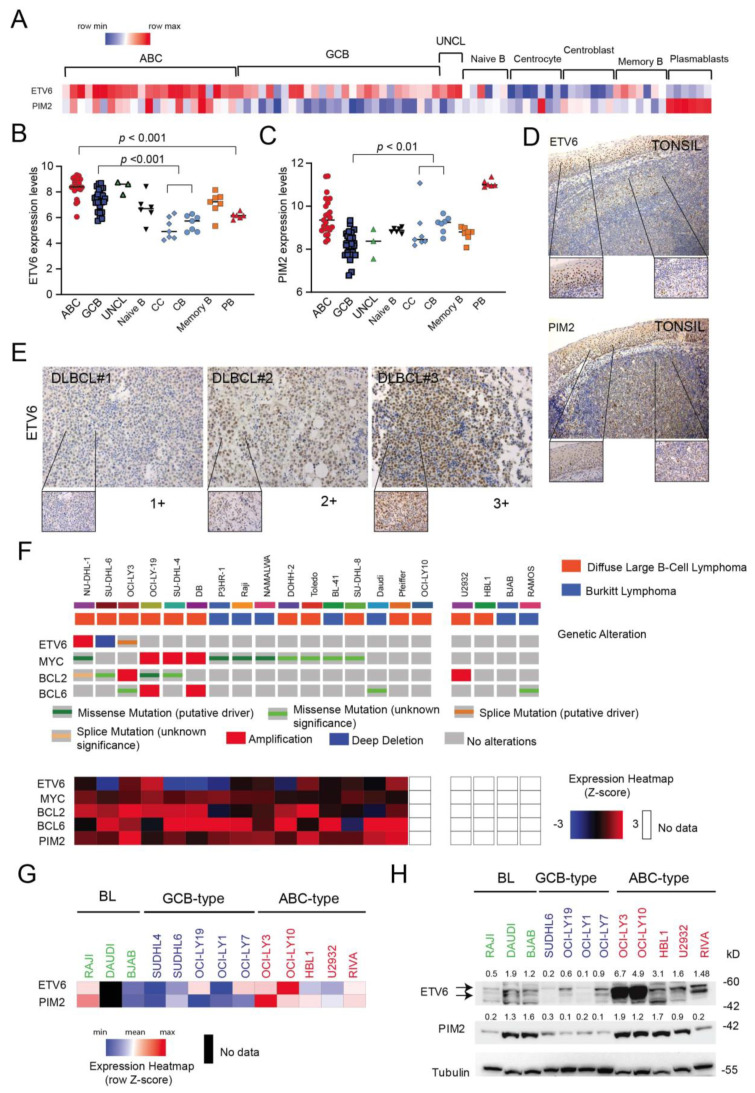
ETV6 expression in malignant B-cells compared to normal B-cell subsets. (**A**) Heat map representation of ETV6 and PIM2 transcript levels in DLBCL samples and normal tonsil B-cell subsets [29]. (**B**) Comparison of ETV6 transcript levels between DLBCL subgroups (ABC, GCB and UNCL) and normal B-cell subsets obtained from tonsils. For statistical analysis, an unpaired *t*-test was used. (**C**) Comparison of PIM2 transcript levels between DLBCL subgroups (ABC, GCB and UNCL) and normal B-cell subsets obtained from tonsils. CC = centrocytes; CB = centroblasts; PB: plasmablasts. For statistical analysis, a nonparametric *t*-test was used. (**D**) ETV6 immunohistochemical staining of reactive tonsil (top): original magnification (×10) and 40× (insets). PIM2 immunohistochemical staining of reactive tonsil (top): original magnification (×10) and 40× (insets). (**E**) Immunohistochemical staining for ETV6 in representative cases of DLBCL patients showing low (+1), medium (+2) and high (+3) expression levels. Original magnification ×20; inset ×40. (**F**) OncoPrint visualization in cBioPortal of genetic alterations (top) affecting *ETV6*, *MYC*, *BCL2* and *BCL6* genes for selected B-lymphoma cell lines (source: Cancer Cell Line Encyclopedia (Broad, 2019) and Lymphoma cell lines (MSKCC,2020)). Heat map showing gene expression levels of selected genes is also depicted (bottom). (**G**) Heat map representation of relative ETV6 and PIM2 transcript levels (normalized to RPL19 housekeeping gene) in available B-cell lymphoma cell lines as determined by qRT-PCR. (**H**) Evaluation of ETV6 and PIM2 and protein expression levels in DLBCL cell lines of different molecular subtypes (ABC and GCB-type) and Burkitt lymphoma (BL) cell lines using immunoblotting. Tubulin is shown as loading control. Relative protein expressions (normalized to loading control) are shown on top of appropriate panels. kD = kilodaltons.

Several lines of evidence suggest that ETV6 may be involved in DLBCL pathogenesis. The *ETV6* gene is altered in approximately 7–10% of DLBCL cases [33], and its alterations (SNV and deletions) are included in the recently defined MYD88 [34], C5 [35] or MCD [36] genomic clusters associated with poor outcome. *ETV6* alterations are more prevalent in the ABC-type DLBCL subgroup [37]. Although *ETV6* is a transcriptional repressor that plays a key role in hematopoiesis and in embryonic development [38], its role in DLBCL and mature B-cells is rather unknown. Data from gene expression analysis of different B-cell subsets [29] indicate that ETV6 transcript levels oscillate between different B-cell stages of development (Figure 5A,B). Indeed, normal germinal center (GC) B-cells (centroblasts, centrocytes) tend to have lower expression levels than B-cells entering (naïve) or exiting the GC reaction (memory and plasmablasts). Furthermore, ETV6 levels seem to increase during oncogenic transformation of GC B-cells to DLBCLs (Figure 5B). This trend is not apparent for PIM2, as oncogenic transformation of GC B-cells seems to be associated with a reduction in PIM2 expression (Figure 5C). IHC analysis executed on reactive tonsils confirmed the rather heterogeneous expression of ETV6 in GC cells compared to malignant B-cells (DLBCL; Figure 5D,E). The above results seem to confirm a role for ETV6 upregulation during B-cell transformation. We subsequently investigated the expression levels of ETV6 and some common genetic alterations in DLBCL and BL cell lines taking advantage of recently available datasets in cBioportal [27,28]. As shown in Figure 5F, ETV6 transcript levels varied across B-cell lines and some alteration of the *ETV6* gene were found including amplifications, deletions and missense mutations (NU-DHL1, SUDHL-6 and OCI-Ly3, respectively). We confirmed differential expression of ETV6 in the DLBCL and BL cell lines by qRT-PCR and Western blot analysis (Figure 5G,H), with ABC-type DLBCL cell lines expressing higher levels of ETV6 and PIM2 compared to GCB-type cell lines.

### 3.6. Inactivation of ETV6 Is Highly Cytotoxic to Aggressive B-Cell Lymphoma Cells

**Figure 6:**
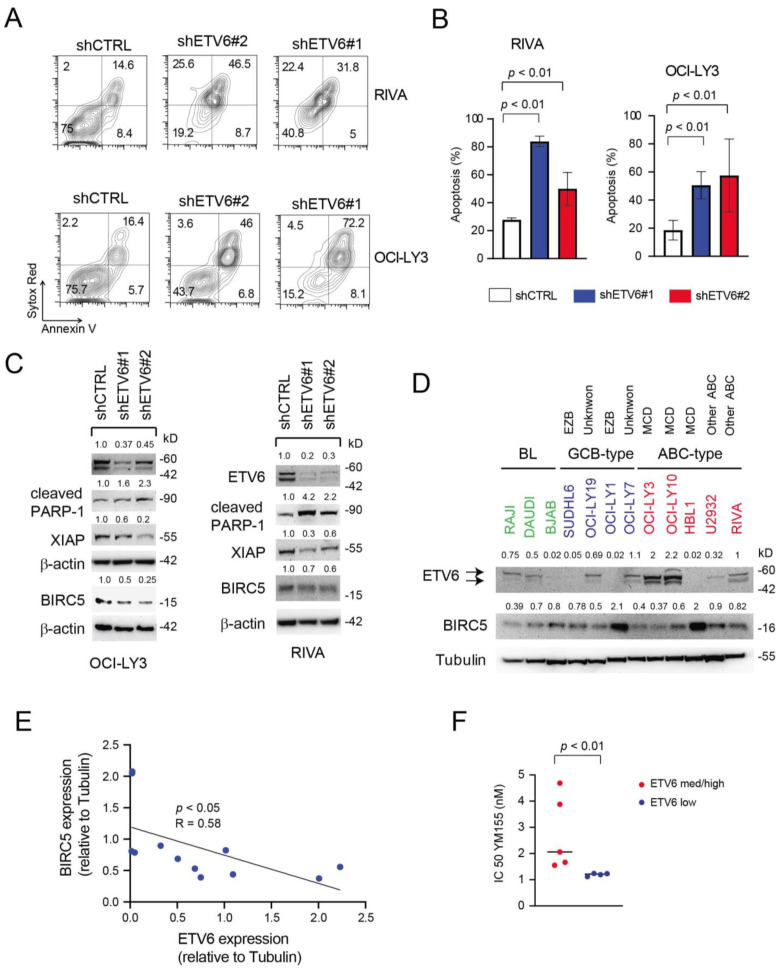
*ETV6* silencing has an important cytotoxic effect in aggressive B-cell lymphoma cell lines. (**A**,**B**) Representative plots of apoptosis (**A**) and quantification of apoptosis (**B**) in RIVA and OCI-Ly3 ABC-type cells (top and bottom or left and right, respectively) transduced with shCTRL, shETV6#1 and shETV6#2 shortly after puromycin selection and maintained for 48 h in regular culture medium (20% FCS). In (**B**), data are represented as Mean ± SD of two independent experiments. For statistical analysis, an unpaired *t*-test was used. (**C**) Western blot evaluating the expression levels of cleaved PARP-1, XIAP and Survivin/BIRC5 in OCI-Ly3 and RIVA cells transduced with shCTRL, shETV6#1 and shETV6#2. β-actin was used as loading control. Relative protein expressions (normalized to loading control) are shown on top of appropriate panels. (**D**) Evaluation of ETV6 and BIRC5 protein expression levels in DLBCL cell lines of different molecular subtypes (ABC and GCB-type) and Burkitt lymphoma (BL) cell lines using immunoblotting. Tubulin is shown as loading control. The numbers above the blots indicate expression levels relative to the loading control. Genetic subgroups [36] of the cell lines are also shown. EZB = *EZH2* mutations and *BCL2* translocations, MCD = *MYD88* and *CD79b* mutations. kD = kilodaltons. (**E**) Linear regression analysis of the relationship between ETV6 and BIRC5 protein levels in B-cell lymphoma cell lines shown in (**D**). (**F**) Dot plot showing the inhibitory concentration determining 50% loss of viability (IC50) for the BIRC5 inhibitor YM155 in relation to ETV6 protein expression levels in B-cell lymphoma cell lines (*n* = 9). For statistical analysis, an unpaired *t*-test was used.

Given the unknown function of ETV6 in mature B-cell lymphomas and our results suggesting that high ETV6 expression may be relevant for the pathogenesis of highly aggressive DLBCL cases, we performed knock-down (KD) experiments in numerous cell lines expressing significant levels of ETV6, such as OCI-Ly3, RIVA, U2932 (all ABC-type), OCI-Ly19 (GCB-type) and RAJI (BL). To this end, cell lines were transduced with two different hairpins targeting *ETV6* (shRNA ETV6#1 and shRNA ETV6#2) and an untargeted hairpin (shRNA CTRL) as control. We found that ETV6 knockdown (KD) determined a marked increase in apoptosis in all cell lines tested (Figure 6A,B and Figure S5A,B). The increase in apoptotic cells (AnnexinV^+^ ± Sytox Red^+^) was associated with increased PARP-1 cleavage (Figure 6C) and reduced expression of anti-apoptotic proteins such as survivin (BIRC5) and XIAP (Figure 6C and Figure S5C).

Apoptosis is a key trait deregulated in cancer and is regulated by two families of proteins: the B-cell leukemia/lymphoma 2 (BCL2) family and the inhibitor of apoptosis protein (IAP) family. Survivin/BIRC5 is a member of the IAP family, and its overexpression has been reported in some studies to be associated with inferior OS in DLBCL patients [39]. Furthermore, YM155 [40], a selective BIRC5 inhibitor, has been found to possess potent antitumor activities against numerous cancers including DLBCL xenografts [41,42] and can determine objective responses in some DLBCL patients as a single agent [43]. Since we found BIRC5 modulation following ETV6 depletion, we screened a panel of DLBCL cell lines for the expression levels of BIRC5 and ETV6 proteins (Figure 6D). Interestingly, we found an inverse relationship between them (Figure 6D,E), suggesting that ETV6 may modulate BIRC5 levels. As no ETV6 specific inhibitor exists and given the recent interest in targeting apoptosis modulators in DLBCL [44], we evaluated the sensitivity our B-lymphoma cell lines to the BIRC5 inhibitor, YM155. We found that cells expressing medium/high levels of ETV6 protein (OCI-Ly19, OCI-Ly10, OCI-Ly3, RIVA and RAJI) were significantly more resistant to YM155 (Figure 6F) compared to cell lines expressing low levels of ETV6 (SUDHL4, HBL1, U2932 and SUDHL6). These results seem to imply that targeting IAPs such as BIRC5 could be most effective in ETV6 low expressing aggressive B-cell malignancies.

## 4. Discussion

DLBCL is the most common type of B-cell lymphoma in the Western world with cure rates of approximately 60% by modern immune chemotherapy (R-CHOP). The remaining 40% of patients experience refractory/relapsing disease and usually succumb to the disease [33,45]. The marked heterogeneity of DLBCL prognosis represents a continuous challenge to physicians and requires the identification of better treatments and outcome predictors either before or shortly after treatment initiation. In particular, it is imperative to identify poor-risk patients in order to offer them more effective therapies. Initial molecular GEP studies revealed that histologically uniform lymphoma subtypes are prognostically and molecularly heterogeneous and identified two biologically distinct groups on the basis of their COO: GCB-like and ABC-like subtypes [6,46]. An additional small subgroup could not be classified into these entities (UNCL) [47]. However, the failure of numerous clinical trials of targeted therapies selecting patients using COO implies that this classification, although highly useful, lacks sufficient granularity to serve any prognostic-therapeutic purpose per se. We tried to address this issue by a different perspective. The aim of our study was to identify a small group of proteins for which its expression predicts survival in patients with DLBCL and that can be readily measured using standard FFPE tissue. Univariate Cox analysis identified many proteins significantly associated with survival, including immune check-point molecules (PDCD1, PDCD2 and PD1L2), components of the PI3K-AKT-mTOR signaling pathway (PTEN, p85 regulatory subunit α), Toll-like receptor signaling (MYD88), JAK-STAT signaling (JAK2), BCR signaling (BLNK) and BCL2, suggesting that these may represent useful therapeutic targets in poor risk patients. However, multivariate Cox analysis disclosed that only PIM2 and ETV6 were independent prognostic factors in our cohort. PIM2 transcript is present in the ABC-signature and is linked to B-cell survival pathways, such as those involving cytokines (IL6, IL10 and IL13) and CD40, NFkB and p53 signaling [48] (see Figure 2). PIM kinases (especially PIM2) have already been proposed as therapeutic targets in DLBCL, especially in ABC-DLBCL cases with aggressive behavior after R-CHOP treatment [48,49]. Unlike these reports, we found that lower levels of PIM2 protein may be associated with reduced OS. This discrepancy may be due to differences in detection methods and cutoff definitions.

What was rather unexpected were also findings regarding ETV6, which is an ETS family transcriptional factor with a crucial role in hematopoiesis and embryonic development [50]. While *ETV6* is frequently rearranged or fused with other genes in human myeloid and lymphoid leukemias [51,52], it is only rarely altered in B-cell lymphoma [53,54]. Recently, however, whole-exome sequencing studies have found a significant fraction of DLBCL samples (mainly of the ABC-subtype) harboring *ETV6* mutations/alterations [35,55]. More precisely, alterations of *ETV6* are considered part of the MCD [36], C5 [35] and MYD88 [34] genetic subgroups associated with poor prognosis. Generally, *ETV6* is inactivated early during leukemogenesis and is considered a tumor suppressor gene [38]. However, most of the mutations found in DLBCL have not been functionally classified (Figure S5D), and *ETV6* alterations do not generally impact ETV6 expression levels [56]. Furthermore, integrated bioinformatics analysis identified *ETV6* as one of the hub genes associated with the two DLBCL subtypes [32]. Thus, the functional role and transcriptional pathways downstream of ETV6 in DLBCL are currently unknown. We found that high ETV6 protein (and transcript) levels are associated with poor survival in B-cell malignancies, especially DLBCLs. Furthermore, from our initial in silico analysis it seems that ETV6 may play a stronger prognostic role in DLBCL NOS compared to PMBL.

ETV6 KD experiments in ABC-type, GCB-type and BL cell lines consistently determined loss of viability, suggesting that acute depletion of ETV6 is highly cytotoxic. Although we do not elucidate the apoptotic program elicited following abrupt ETV6 loss, this event was often associated with BIRC5 protein depletion. Of note, ETV6 depleted cells that persisted in vitro showed a paradoxical increase in BIRC5 expression (data not shown), suggesting that only abrupt ETV6 loss is cytotoxic. Studying the relationship between ETV6 and BIRC5 in established high-grade B-cell lymphoma cell lines revealed an inverse relationship between these two proteins. Interestingly, we found numerous putative ETV6 binding sites (GGAA/T) in the *BIRC5* promoter region, suggesting that ETV6 may regulate its expression. Consistently, cell lines exhibiting high ETV6 protein levels were found to be more resistant to the BIRC5 inhibitor, YM155. The small molecule YM155, although seemingly well tolerated, as a single-agent has demonstrated only limited activity in refractory DLBCL patients in a phase II clinical trial [57]. More encouraging results, however, have been obtained by the combination with rituximab or bendamustine in preclinical models [58]. Our study suggests that evaluation of ETV6 (possibly in combination with PIM2 to determine the ETV6/PIM2 ratio) may serve two purposes, as high ETV6 levels may correlate with poor prognosis, while low ETV6 levels may identify DLBCL patients, who could benefit from a different therapeutic intervention (e.g., inclusion of BIRC5 inhibitors, such as YM155).

Our study has limitations that may impact the obtained results and that need to be taken into account. These include its retrospective nature, the small sample size (*n* = 47), its histological and therapeutic heterogeneity and problems related to COO stratification in a subset of samples. Thus, further clinical investigation of the prognostic significance of ETV6 expression needs to be performed in a larger prospective series of uniformly treated DLBCL patients, ideally in combination with targeted sequencing analyses [34]. Furthermore, the tolerability and therapeutic potential of combination regimens incorporating YM155 in selected patients awaits testing.

## 5. Conclusions

By using antibody arrays, we identified a subgroup of aggressive DLBCLs, characterized by high ETV6 and low PIM2 protein levels (or high ETV6/PIM2 ratio by IHC). Elevated ETV6 transcript levels were also found to be associated with adverse clinical outcomes. By using KD experiments, we demonstrate that ETV6 may have an oncogenic function and that high ETV6 expressing cells are more resistant to BIRC5 inhibition. Conversely, low ETV6 expression may identify B-lymphoma patients, who may benefit from the addition of YM155 (a BIRC5 inhibitor) to their treatment regimen.

## Figures and Tables

**Table 1:** Clinical characteristics of evaluated patients.

Patients and Tumor Characteristics			
Features	CR (33)	PR or PD (Less than CR; 14)	*p* Value
Median age, range	67 (20–83)	73 (47–82)	n.d.
Male sex	17 (51%)	7 (50%)	0.93
AAS III-IV	18 (55%)	10 (71%)	0.28
Elevated LDH	11 (33%)	8 (57%)	0.13
ECOG PS ≥ 2	3 (9%)	6 (43%)	0.007
Extra nodal site ≥ 2	2 (6%)	3 (21%)	0.12
Bulky > 6 cm	12 (36%)	5 (36%)	0.96
IPI risk group			0.019
Low (0–1)	17 (52%)	3 (9%)	
Intermediate (2–3)	14 (42%)	6 (43%)	
Poor (4–5)	2 (6%)	5 (36%)	
Histology			n.d.
DLBCL NOS	31 (94%)	13 (93%)	
DLBCL CNS	2 (6%)	1 (7%)	
Cell of Origin (Hans)			0.62
GCB	12 (36%)	5 (35%)	
Non GCB	21 (63%)	6 (43%)	
Not evaluated	0	3 (21%)	
CGA			0.0015
Fit	15 (45%)	1 (7%)	
Unfit	4 (12%)	4 (28%)	
Frail	1 (3%)	5 (36%)	
Not assessed	13 (39%)	4 (29%)	
Primary treatment			n.d.
R-CHOP/R-COMP/COMP/R-AC/R-CHOP + HD MTX	27	8	
R-Bendamustine	0	1	
R-CVP	1	3	
MTX/ARA-C ± R	2	1	
R-VACOP-B	2	1	
R-Hyper CVAD	1	0	
Alive at last follow-up	24 (72%)	1 (7%)	<0.0001

CR: Complete Remission, PR: Partial Response, PD: Progressive Disease, AAS: Ann Arbor Staging System, LDH: Lactate dehydrogenase, *ECOG PS*: Eastern Cooperative Oncology Group Performance Status, IPI: International Prognostic Index, DLBCL NOS: Diffuse Large B-cell Lymphoma Not otherwise Specified, HD = High dose, CNS: Central Nervous System, n.d.: not determined.

**Table 2:** Proteins significantly related to OS (*p* < 0.05) in a univariate Cox analysis.

UniProtKB	Protein Name	Regression Coefficient	*p*-Value
Q9HC98	NEK6	9.9051	0.005
O15181	CD21	−3.3531	0.0089
P27986	P85A	−8.8588	0.0008
Q02548	PAX5	−0.914	0.0475
Q15116	PDCD1	13.5509	0.0004
Q16342	PDCD2	7.034	0.0013
P00558	PGK1	7.2112	0.0008
Q9NWQ8	PAG1	−16.0977	0.0004
Q9P1W9	PIM2	−1.1727	0.0062
P60484	PTEN	3.8517	0.0006
P62937	PPIA	−1.2619	0.0647
P18031	PTPN1	−4.3549	0.0018
Q9BQ51	PD1L2	−1.9748	0.0027
P55895	RAG2	−3.035	0.0008
P06703	S100A6	−11.2893	0.0029
Q9H334	FOXP1	5.85	0.0034
P04406	G3P/GAPDH	−3.6582	0.0002
P05112	IL4	−4.8767	0.0022
P05231	IL6	4.8604	0.0011
P27987	IP3KB/ITPKB	0.9472	0.0514
Q15306	IRF4	−3.2923	0.0097
O60674	JAK2	1.35	0.0318
Q9UGP4	LIMD1	3.15	<0.0001
O60449	LY75/DEC-205	−10.7107	0.0005
P10243	MYBA/MYBL1	2.0831	0.0959
Q99836	MYD88	−3.5678	0.0047
O60239	3BP5/SH3BP5	6.0439	0.0003
P61769	B2MG	−0.8181	0.0992
P10415	BCL2	3.0367	0.0106
P08236	BGLR/GUSB	−2.6631	0.0287
Q8WV28	BLNK	2.4548	0.0011
Q92583	CCL17/TARC	6.7439	0.0014
P10147	CCL3	4.1361	0.0105
P32248	CCR7	−3.5845	<0.0001
P26842	CD27	−6.0218	0.0064
P60033	CD81	7.0502	0.0006
P49715	CEBPA	4.0571	0.0527
P04141	CSF2/GMCSF	−10.0918	0.0032
P29279	CTGF/IGFBP-8	−7.2609	0.0138
P49961	ENTP1/CD39	−4.3636	0.0176
P41212	ETV6/TEL1	1.6527	0.0017

**Table 3:** Proteins selected as prognostic factors in the stepwise multivariate survival analysis performed by Cox proportional hazards model.

UniProtKB	Protein Name	HR (95% CI)	*p*-Value	Regression Coefficient
P41212	ETV6/TEL1	1.09–3.43	0.0286	0.6489
Q9P1W9	PIM2	0.57–0.98	0.0346	−0.2927

**Table 4:** Multivariate survival analysis of the ETV6-PIM2 protein score with OS as dependent variable in our cohort of DLBCL patients.

Variable	Wald Statistic (Z-Score)	*p*-Value	Regression Coefficient (b)	Exp(b)	95% CI of Exp(b)
IPI score versus	6.28	0.012	0.411	1.5083	1.09–2.08
ETV6-PIM2 score	4.19	0.04	1.01	2.76	1.04–7.28

## Data Availability

The study utilized, in part, publicly available datasets (Gene Expression Omnibus, cBioportal and GeneMANIA). Antibody array data will be made available upon reasonable request to the authors.

## Supplementary Materials

The following are available online at https://www.mdpi.com/article/10.3390/cancers14020338/s1, Supplementary Materials and Methods, Table S1: List of proteins targeted by antibodies present in our arrays. Figure S1: Comparative Marker Selection analysis to identify differentially expressed proteins between DLBCL not otherwise specified (DLBCL NOS) and primary central nervous system DLBCL (DLBCL CNS) samples. Figure S2: Research design and representation of proteins associated with survival. Figure S3: Concordance of ETV6 and PIM2 levels between protein arrays and immunohistochemistry. Figure S4: Prognostic role of ETV6 transcript levels in DLBCL patients. Figure S5: Silencing of ETV6 has a cytotoxic effect on B lymphoma cells.
