# Supplementary material for: High ETV6 Levels Support Aggressive B Lymphoma Cell Survival and Predict Poor Outcome in Diffuse Large B-Cell Lymphoma Patients
Source: Cancers (Basel). 2022 Jan 11;14(2):338. doi: 10.3390/cancers14020338 (PMC8774128; doi:10.3390/cancers14020338)
Supplement: Supplementary file 1 [file cancers-14-00338-s001.zip › cancers-1508138-supplementary/cancers-1508138-supplementary.pdf]

cancers

MDPI

Article

High ETV6 Levels Support Aggressive B Lymphoma Cell Survival and Predict Poor Outcome in Diffuse Large B-Cell Lymphoma Patients

Dario Marino [1,†], Marco Pizzi [2,†], Iuliia Kotova [3], Ronny Schmidt [3], Christoph Schröder [3], Vincenza Guzzardo [2], Ilaria Talli [4], Edoardo Peroni [5], Silvia Finotto [1], Greta Scapinello [6], Angelo Paolo Dei Tos [2], Francesco Piazza [6], Livio Trentin [6], Vittorina Zagonel [1] and Erich Piovan [4,5,*]

SUPPLEMENTARY MATERIAL

-Supplementary Materials and Methods

-Supplementary References

-Supplementary Table S1

-Supplementary Figure S1

-Supplementary Figure S2

-Supplementary Figure S3

-Supplementary Figure S4

-Supplementary Figure S5

S1. Supplementary Materials and Methods

S1.1. Cell Lines

The following lymphoma cell lines were used in this study: Burkitt lymphoma (BL) cell lines (RAJI, DAUDI and BJAB) were obtained from Dr. M. Ferrarini (National Cancer Research Institute, Genoa, Italy), DLBCL cell lines OCI-LY1, OCI-LY7, OCI-LY3, OCI-LY10, HBL1, U2932, SUDHL4 and SUDLH6 (generous gifts from Riccardo Dalla Favera, Columbia University, USA). Cells were cultured in RPMI-1640 medium or IMDM medium (Euroclone, Pero, Italy) supplemented with 10% to 20% fetal bovine serum (FBS; Gibco, Thermo Fisher Scientific, Waltham, MA, USA) at 37 °C under 5% CO_2.

S1.2. Array Production, Sample Labeling, Hybridization and Data Analysis

Only antibodies for which validation data using different techniques such as Western Blot, IHC or other data such as usability in ELISA or Western Blot was available from the manufacturer or provider, were used for the microarrays. All molecules were immobilized by contact printing on epoxysilane slides (Schott Nexterion, Jena, Germany) at a fixed concentration, using a MicroGrid robot (BioRobotics, Cambridge, UK) and SMP3B pins (Telechem, Sunnyvale, USA). All arrays used in this study were from a single production batch of more than 250 microarrays. All antibodies were spotted eight times in a randomized pattern in different array sectors. After printing, the slides were kept at 4°C in a humidity-free environment until use. FFPE tissue samples (5 sections of 5-8 µm each) were extracted according to Sciomics standard operating procedures (SOP). The bulk protein concentration was determined by BCA assay. All samples fulfilled the quality criteria for an analysis.

After a 3 hour incubation, slides were washed and subsequently dried with nitrogen before being scanned using a Powerscanner (Tecan, Austria). All microarrays were scanned with identical instrument laser power and adjusted PMT settings. Acquired raw data were analysed using the linear models for microarray data (LIMMA) [1] package of R-Bioconductor after uploading the median signal intensities. For normalization, a specialized invariant Lowess method was applied [2], taking advantage of the always present reference sample. For differential analysis of protein expression, a one-factorial linear model was fitted with LIMMA resulting in a two-sided t-test based on moderated statistics. All P-values were adjusted for multiple testing by controlling the false discovery rate (FDR) according to Benjamini and Hochberg [3]. For hierarchical clustering, the heatmapper software [4] and Genepattern software [5] were used for visualizing agglomeration using the pearson distance measurement and the average linkage algorithm. Comparative Marker selection (Genepattern) was also performed. Heat maps were also generated using GraphPad Prism (GraphPad Software, San Diego, CA, USA).

S1.3. Nanostring Assay for COO

Total RNA was extracted from FFPE tissue material using RNeasy FFPE kit (Qiagen, Hilden, Germany) according to the manufacturer's protocol. After quantification with Qubit RNA assay kit (Life Technologies), samples were subjected to the Lymph2Cx gene

expression assay (Nanostring Technologies, Seattle, WA, USA) according to the manufacturer's instructions.

S1.4. Lysate Preparation for Western Blotting

Total cell lysates from DLBCL cell lines were prepared using RIPA lysis buffer supplemented with phosphatase inhibitor cocktail set I and II (Sigma-Aldrich, Saint Louis, MO) and protease inhibitor cocktail tablets (Roche, Burgess Hill, UK) and normalized for protein concentration using the BCA method (Pierce, Pero, Italy). For DLBCL primary samples, FFPE material (5 sections of 5-8 μm each) was used to extract and isolate proteins according to the Qproteome FFPE kit (Qiagen, Hilden Germany) or using the Sciomics SOP. The bulk protein concentration was determined by BCA assay.

S1.5. Quantitative Real Time RT-PCR

Total RNA was extracted using TRIzol Reagent (Invitrogen) following manufacturer instructions and cDNA was synthesized from 0.5-1 μg of total RNA using SensiFAST™ cDNA Synthesis Kit (Bioline, London, UK). Real time PCR reactions were performed using SensiFAST™ SYBR® Hi-ROX Kit (Bioline) and run on an ABI Prism 7900 Sequence Detection System (Applied Biosystems, Thermo Fisher Scientific). Relative gene expression levels were calculated using the $2^{-\Delta\Delta Ct}$ method [6] and normalized against the expression of *RPL19* housekeeping gene. Primer sequences used for Real time RT-PCR reactions are available upon request.

References

1. Smyth, G. K., Linear models and empirical bayes methods for assessing differential expression in microarray experiments. *Statistical applications in genetics and molecular biology* **2004,** 3, Article3.
2. Sill, M.; Schroder, C.; Hoheisel, J. D.; Benner, A.; Zucknick, M., Assessment and optimisation of normalisation methods for dual-colour antibody microarrays. *BMC bioinformatics* **2010,** 11, 556.
3. Reiner, A.; Yekutieli, D.; Benjamini, Y., Identifying differentially expressed genes using false discovery rate controlling procedures. *Bioinformatics* **2003,** 19, (3), 368-75.
4. Babicki, S.; Arndt, D.; Marcu, A.; Liang, Y.; Grant, J. R.; Maciejewski, A.; Wishart, D. S., Heatmapper: web-enabled heat mapping for all. *Nucleic acids research* **2016,** 44, (W1), W147-53.
5. Reich, M.; Liefeld, T.; Gould, J.; Lerner, J.; Tamayo, P.; Mesirov, J. P., GenePattern 2.0. *Nature genetics* **2006,** 38, (5), 500-1.
6. Livak, K. J.; Schmittgen, T. D., Analysis of relative gene expression data using real-time quantitative PCR and the 2(-Delta Delta C(T)) Method. *Methods* **2001,** 25, (4), 402-8.
7. Dubois, S.; Tesson, B.; Mareschal, S.; Viailly, P. J.; Bohers, E.; Ruminy, P.; Etancelin, P.; Peyrouze, P.; Copie-Bergman, C.; Fabiani, B.; Petrella, T.; Jais, J. P.; Haioun, C.; Salles, G.; Molina, T. J.; Leroy, K.; Tilly, H.; Jardin, F., Refining diffuse large B-cell lymphoma subgroups using integrated analysis of molecular profiles. *EBioMedicine* **2019,** 48, 58-69.

Table S1. List of proteins targeted by antibodies present in our arrays.

UniProtKB	Protein name	Sciomics antibody ID
P10721	KIT_HUMAN	sc_001
Q9NZQ7	PD1L1_HUMAN	sc_002
Q15116	PDCD1_HUMAN	sc_003
Q8WV28	BLNK_HUMAN	sc_005
Q15910	EZH2_HUMAN	sc_006
O60674	JAK2_HUMAN	sc_007
P10243	MYBA_HUMAN	sc_008
O43516	WIPF1_HUMAN	sc_009
Q00987	MDM2_HUMAN	sc_010
P25963	IKBA_HUMAN	sc_011
Q9NZQ7	PD1L1_HUMAN	sc_012
P10415	BCL2_HUMAN	sc_013
P10147	CCL3_HUMAN	sc_014
Q92583	CCL17_HUMAN	sc_015
P32248	CCR7_HUMAN	sc_016
O15181	CD21_HUMAN	sc_017
P26842	CD27_HUMAN	sc_018
P49961	ENTP1_HUMAN	sc_019
P16150	LEUK_HUMAN	sc_020
P16070	CD44_HUMAN	sc_021
P60033	CD81_HUMAN	sc_022
P49715	CEBPA_HUMAN	sc_023
P29279	CTGF_HUMAN	sc_024
P10145	IL8_HUMAN	sc_025
P48061	SDF1_HUMAN	sc_026
P62937	PPIA_HUMAN	sc_027
P41212	ETV6_HUMAN	sc_028
P02751	FINC_HUMAN	sc_029
O15519	CFLAR_HUMAN	sc_030
Q9H334	FOXP1_HUMAN	sc_031
Q9BYC5	FUT8_HUMAN	sc_032
P04406	G3P_HUMAN	sc_033
P08236	BGLR_HUMAN	sc_034
P05112	IL4_HUMAN	sc_035
P05231	IL6_HUMAN	sc_036
P22301	IL10_HUMAN	sc_037
Q14005	IL16_HUMAN	sc_038
Q15306	IRF4_HUMAN	sc_039
Q9UGP4	LIMD1_HUMAN	sc_041

UniProtKB	Protein name	Sciomics antibody ID
P25791	LMO2/RBTN2_HUMAN	sc_042
Q12912	LRMP_HUMAN	sc_043
P61769	B2MG_HUMAN	sc_044
P01106	MYC_HUMAN	sc_045
P19838	NFKB1_HUMAN	sc_046
P23510	TNFL4_HUMAN	sc_047
P04637	P53_HUMAN	sc_048
Q9NWQ8	PHAG1_HUMAN	sc_049
Q9BQ51	PD1L2_HUMAN	sc_050
P27986	P85A_HUMAN	sc_051
Q9P1W9	PIM2_HUMAN	sc_052
Q9Y483	MTF2_HUMAN	sc_053
P60484	PTEN_HUMAN	sc_054
P17947	SPI1_HUMAN	sc_055
P09486	SPRC_HUMAN	sc_056
P06703	S10A6_HUMAN	sc_057
Q07011	TNR9_HUMAN	sc_058
P08473	NEP_HUMAN	sc_060
Q99836	MYD88_HUMAN	sc_061
Q9BXL7	CAR11_HUMAN	sc_062
Q9UK53	ING1_HUMAN	sc_063
Q13291	SLAF1_HUMAN	sc_064
Q86WD7	SPA9_HUMAN	sc_065
P00558	PGK1_HUMAN	sc_066
O60449	LY75_HUMAN	sc_067
Q9HC98	NEK6_HUMAN	sc_068
P18031	PTN1_HUMAN	sc_069
P30279	CCND2_HUMAN	sc_070
Q96LC9	BMF_HUMAN	sc_071
Q68CJ6	SLIP_HUMAN	sc_072
P40763	STAT3_HUMAN	sc_073
P38936	CDKN1A_HUMAN	sc_074
Q02763	TIE2_HUMAN	sc_075
O15524	SOCS1_HUMAN	sc_076
P11309	PIM1_HUMAN	sc_077
P55895	RAG2_HUMAN	sc_078
P27987	IP3KB_HUMAN	sc_079
A2RUS2	DEND3_HUMAN	sc_080
Q16342	PDCD2_HUMAN	sc_081
Q8IVM0	CCD50_HUMAN	sc_082

O60239	3BP5_HUMAN	sc_083
**UniProtKB**	**Protein name**	**Sciomics antibody ID**
P04141	CSF2_HUMAN	sc_084
Q02548	PAX5_HUMAN	sc_085

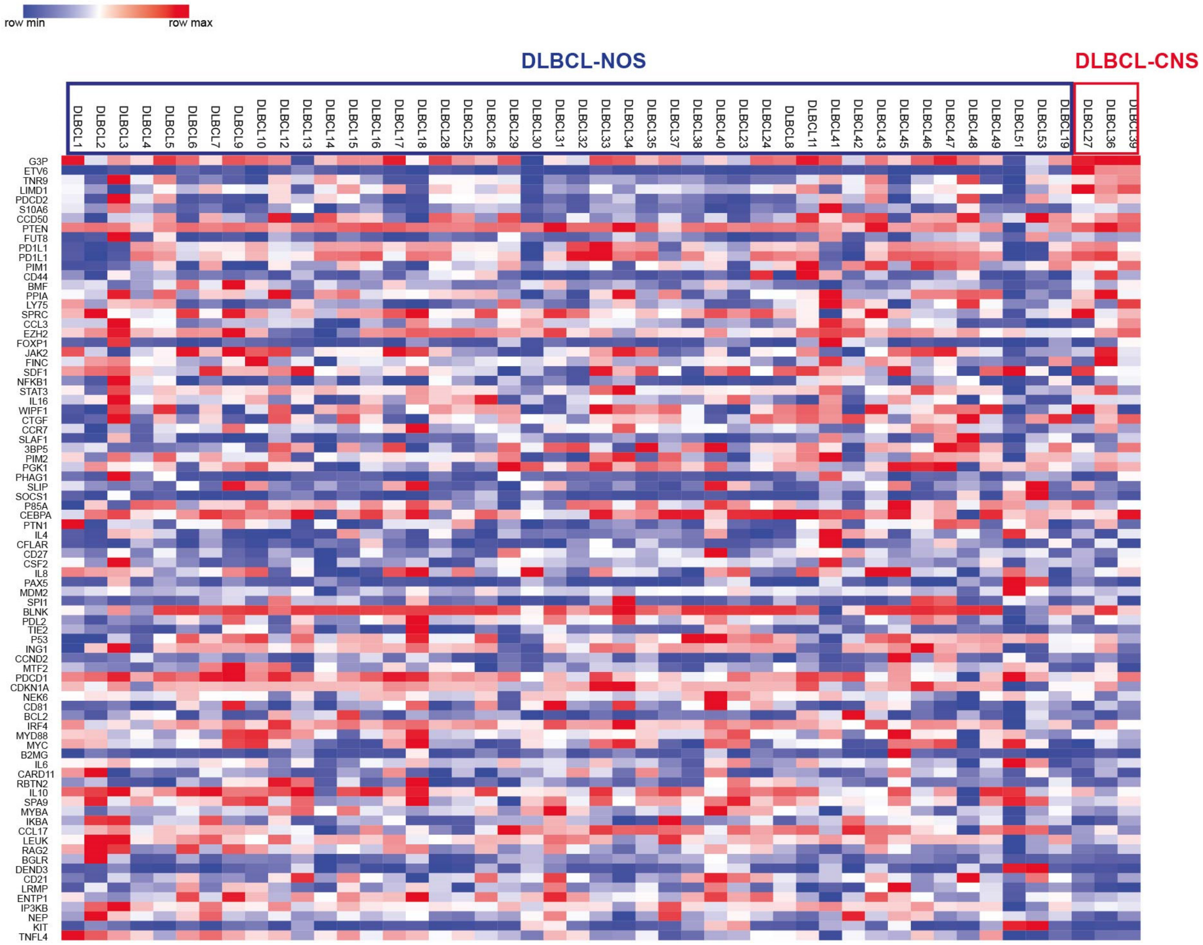

Figure S1. Comparative Marker Selection analysis was performed to identify differentially expressed proteins between DLBCL not otherwise specified (DLBCL-NOS) and primary DLBCL of the central nervous system (DLBCL-CNS) samples.

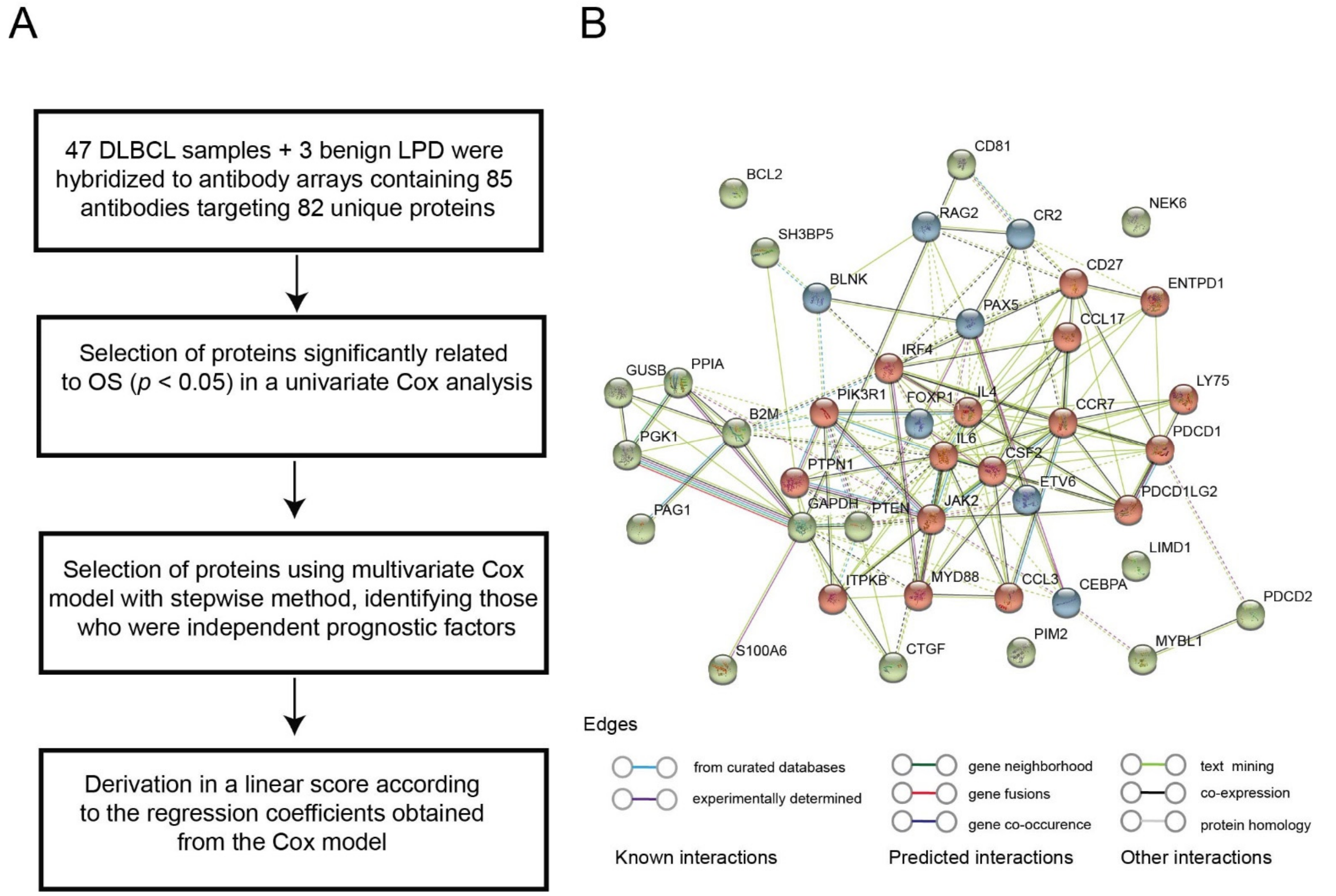

Figure S2. Research design and representation of proteins associated with survival. (**A**) Research design and workflow leading to the identification of proteins associated with survival. LPD= benign reactive lymph nodes. (**B**) Proteins identified as associated with survival through univariate Cox regression analysis. STRING software was used to determine connections between identified proteins. Network nodes represent proteins, while edges represent protein-protein associations. Stronger associations are represented by thicker lines.

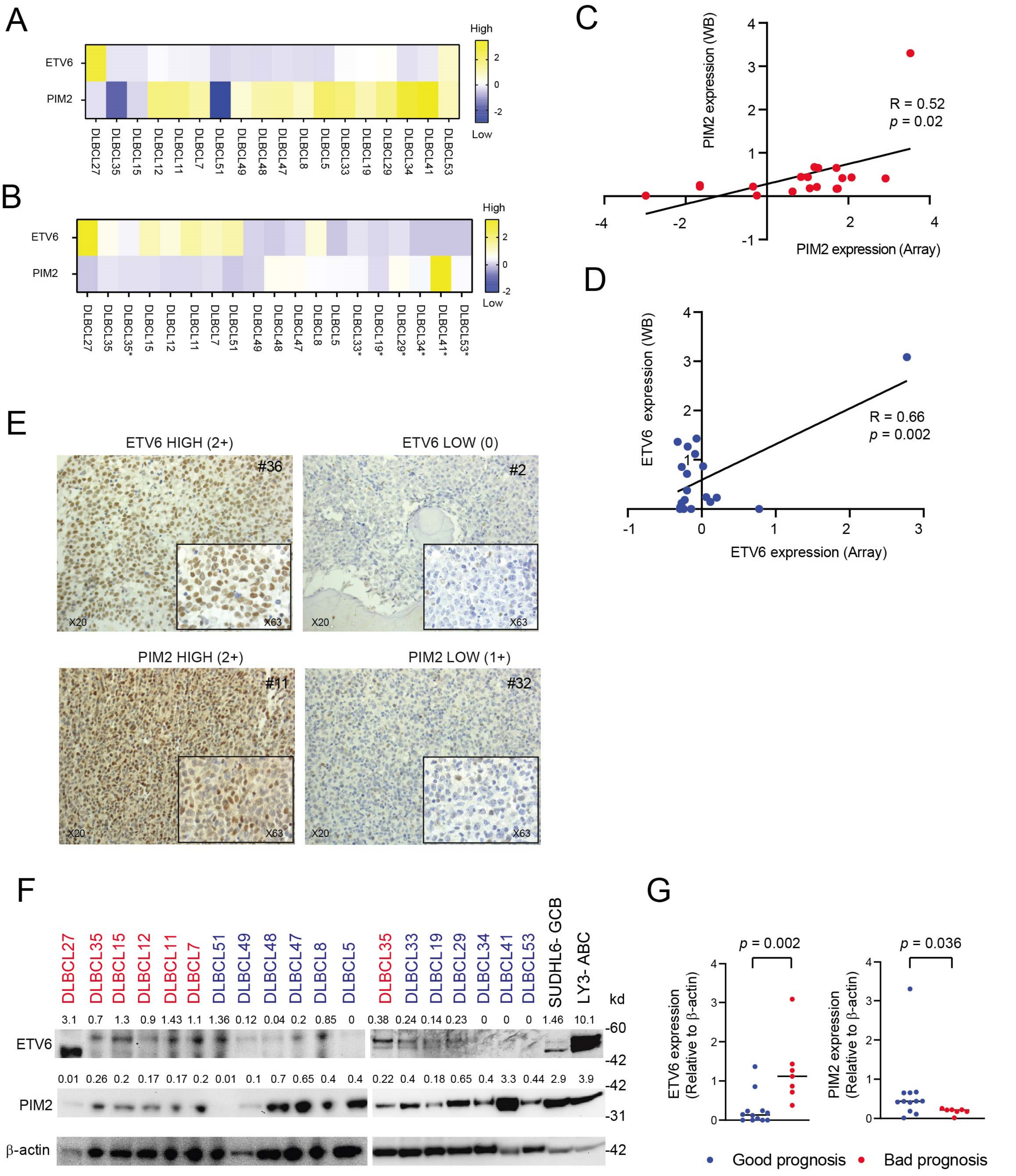

Figure S3. Concordance of ETV6 and PIM2 levels between protein arrays and immunohistochemistry. (**A**) Heat map representation of relative ETV6 and PIM2 protein levels (normalized to β-actin levels) in selected DLBCL samples as determined by immunoblotting. (**B**) Heat map representation of ETV6 and PIM2 protein levels in the same DLBCL samples as (**a**) determined by antibody arrays. (**C**) Linear regression analysis of the relationship between PIM2 expression levels determined by immunoblotting (WB) and antibody arrays. (**D**) Linear regression analysis of the relationship between ETV6 expression levels determined by immunoblotting (WB)

and antibody arrays (right panel). (**E**) Immunohistochemical stainings for ETV6 and PIM2 in representative cases of DLBCL patients showing high (+2 score) and low expression (0/+1 score) of these proteins. Original magnification X20; inset X63. (**F**) Evaluation of PIM2 and ETV6 protein expression levels in selected patients using immunoblotting. β-actin is shown as loading control. Numbers above the blots indicate expression levels relative to the loading control. Samples showing adverse effects (death) are labeled in red. kD= kilodaltons. (**G**) Quantification of ETV6 and PIM2 proteins levels from (**F**). Samples with poor prognosis (labeled in red) have high ETV6 expression and low PIM2 expression. For statistical analysis, an unpaired t-test was used.

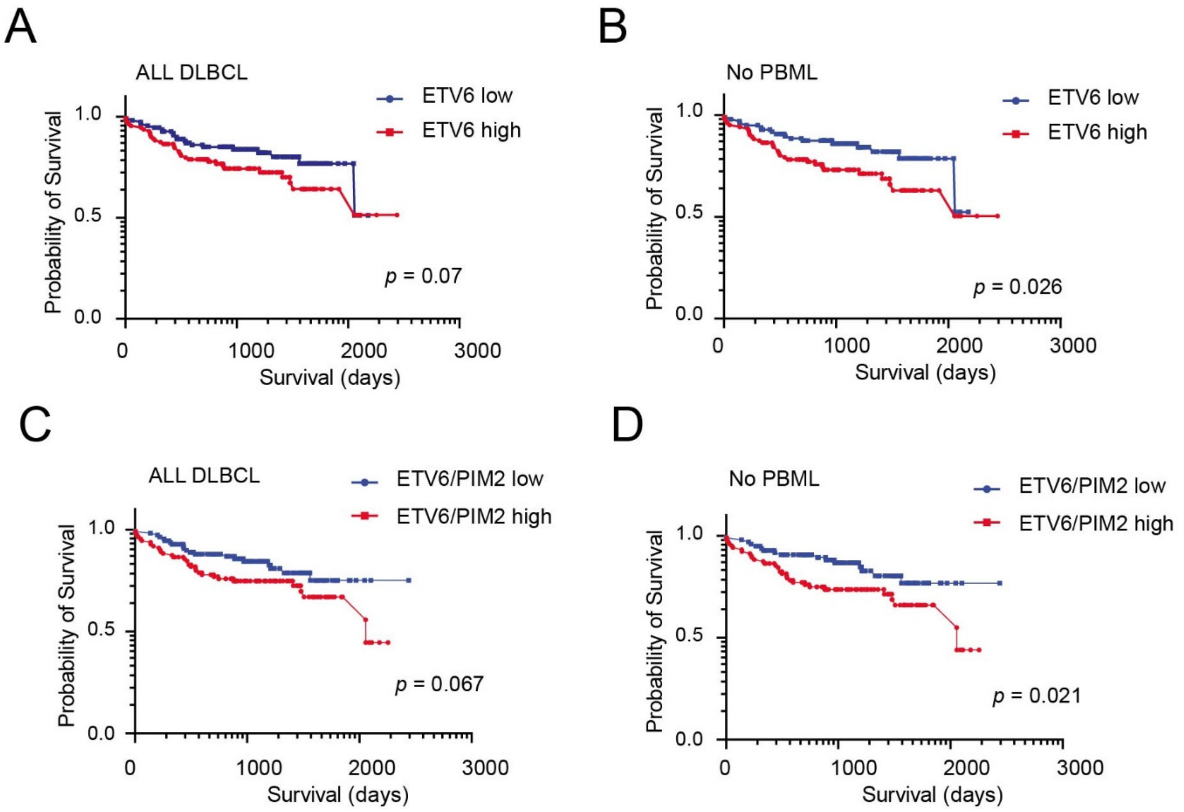

Figure S4. Prognostic role of ETV6 transcript levels in DLBCL patients. (**A**) Kaplan-Meier survival curves of the entire series of 223 DLBCL patients [7], analysed as a group (**A**) or excluding PBML cases (**B**). ETV6 (probe 205585_at) high cases (> median expression) or ETV6 low cases (< median expression). Kaplan-Meier survival curves of the entire series of 223 DLBCL patients [7], analysed as a group (**C**) or excluding PBML cases **(D**). High ETV6/PIM2 ratio (> median level) or low ETV6/PIM2 ratio cases (< median expression).

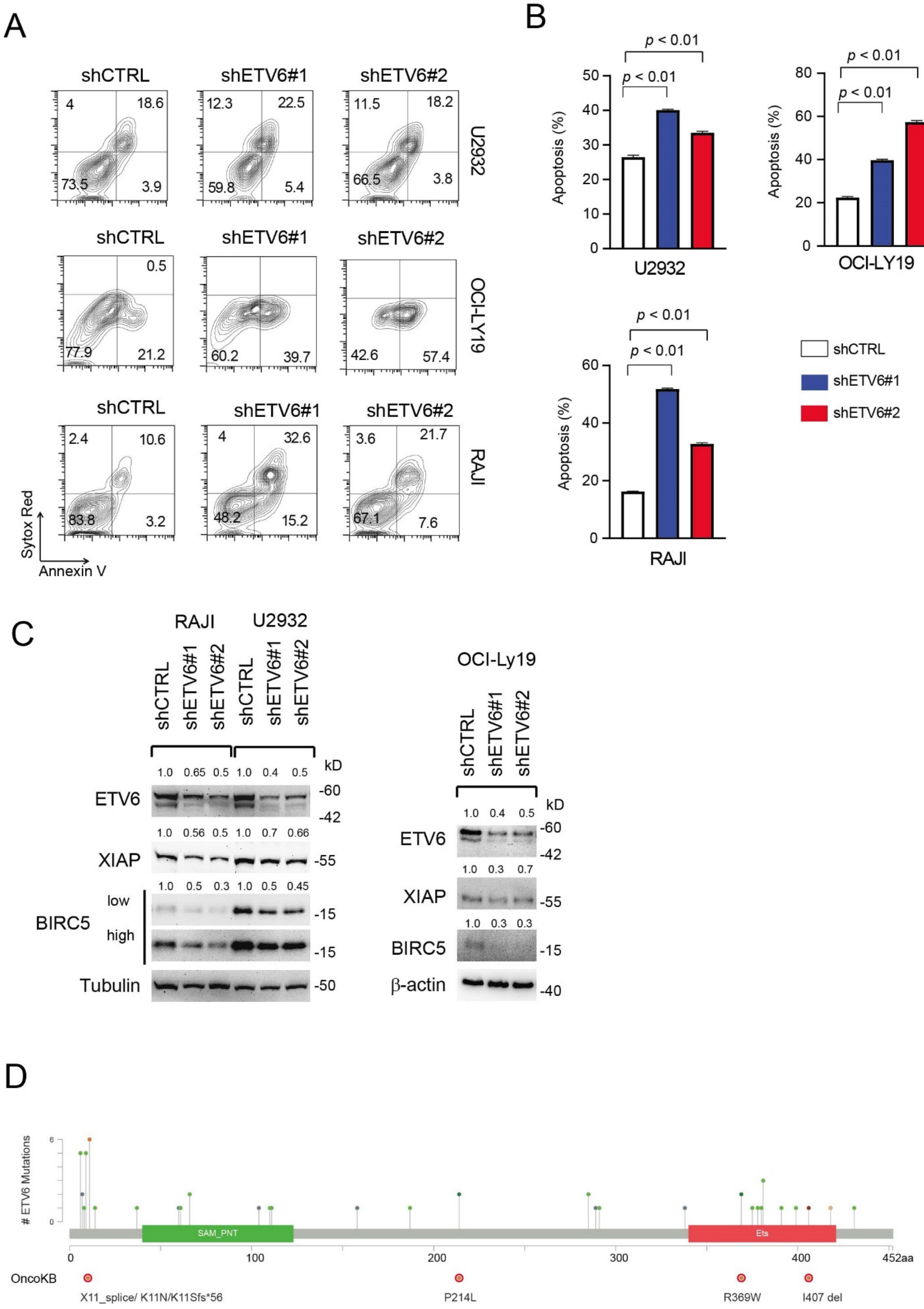
A
shCTRL
shETV6#1
shETV6#2
U2932
4
18.6
73.5
3.9
12.3
22.5
59.8
5.4
11.5
18.2
66.5
3.8
OCI-LY19
0.5
77.9
21.2
60.2
39.7
42.6
57.4
RAJI
2.4
10.6
83.8
3.2
4
32.6
48.2
15.2
3.6
21.7
67.1
7.6
Sytox Red
Annexin V
B
p < 0.01
p < 0.01
Apoptosis (%)
U2932
OCI-LY19
RAJI
shCTRL
shETV6#1
shETV6#2
C
RAJI
U2932
OCI-Ly19
kD
1.0 0.65 0.5 1.0 0.4 0.5
ETV6
-60
-42
1.0 0.56 0.5 1.0 0.7 0.66
XIAP
-55
1.0 0.5 0.3 1.0 0.5 0.45
BIRC5
low
high
-15
Tubulin
-50
1.0 0.4 0.5
1.0 0.3 0.7
1.0 0.3 0.3
β-actin
-40
D
ETV6 Mutations
SAM_PNT
Ets
452aa
OncoKB
X11_splice/ K11N/K11Sfs*56
P214L
R369W
I407 del

Figure S5. Silencing of ETV6 has a cytotoxic effect on B lymphoma cells. (**A,B**) Representative plots of apoptosis (**A**) and quantification of apoptosis (**B**) in U2932 ABC-type, OCI-Ly19 GCB type and RAJI BL cells (from top to bottom, respectively) transduced with shCTRL, shETV6#1, shETV6#2 shortly after puromycin selection and maintained for 48 h in regular culture medium (10 or 20% FCS). The results of one of three experiments performed with similar results are shown. For statistical analysis, an unpaired *t*-test was used. (**C**) Western blot evaluating the expression levels of XIAP, Survivin/BIRC5 and ETV6 in RAJI and U2932 lymphoma cells (left) or OCI-Ly19 cells (right) transduced with shCTRL, shETV6#1, shETV6#2. Tubulin or β-actin was used as loading control. Numbers above the blots indicate expression levels relative to the loading control. (**D**) Lollipop plots of ETV6 mutations found in DLBCL cases (cBioPortal). Putatively oncogenic mutations identified by OncoKB are also shown.
