# Supplementary figures and images for: High ETV6 Levels Support Aggressive B Lymphoma Cell Survival and Predict Poor Outcome in Diffuse Large B-Cell Lymphoma Patients
Source: Cancers (Basel). 2022 Jan 11;14(2):338. doi: 10.3390/cancers14020338 (PMC8774128; doi:10.3390/cancers14020338)

Fig. 5 H

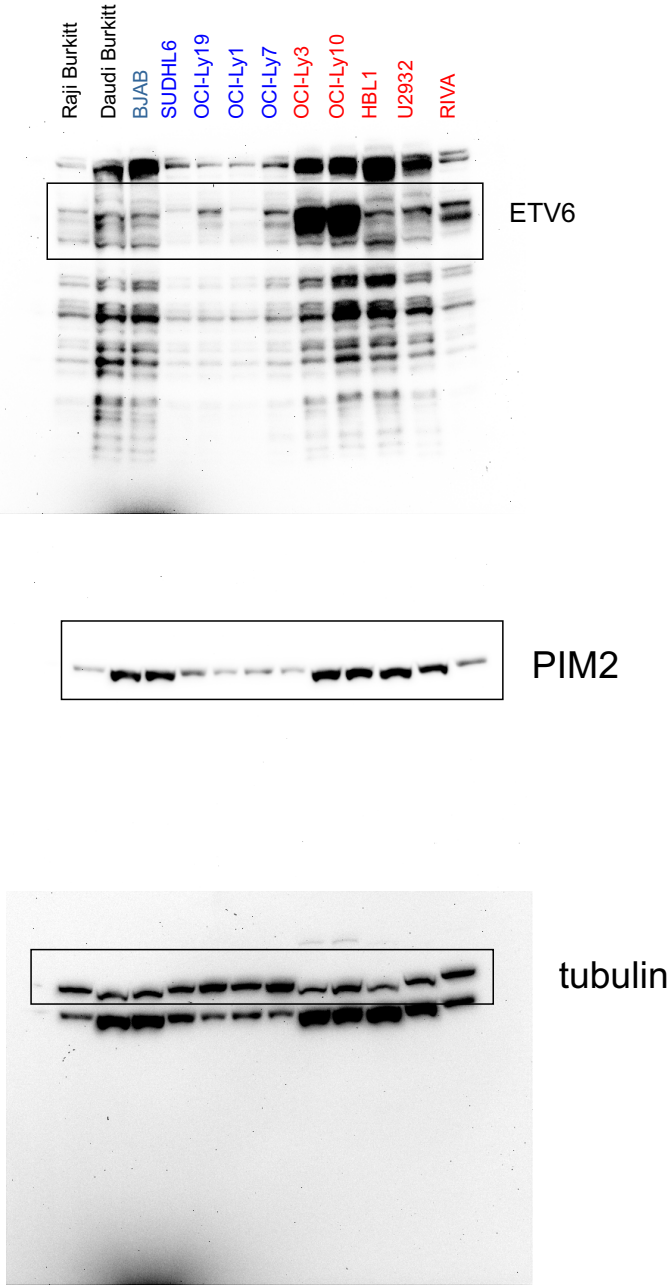

Fig. 6C

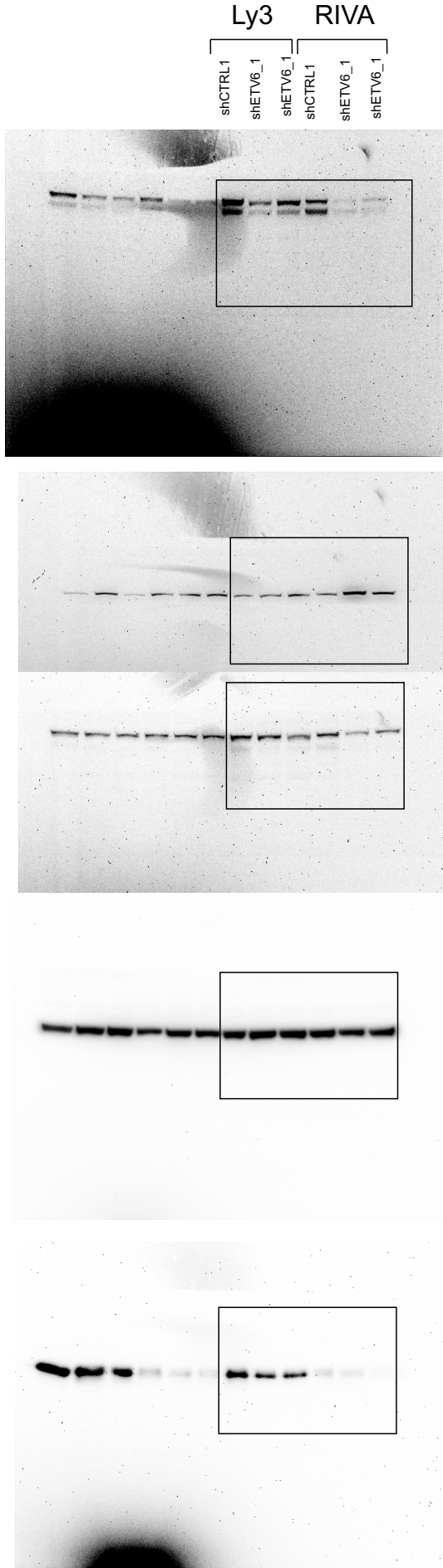

Fig. 6D

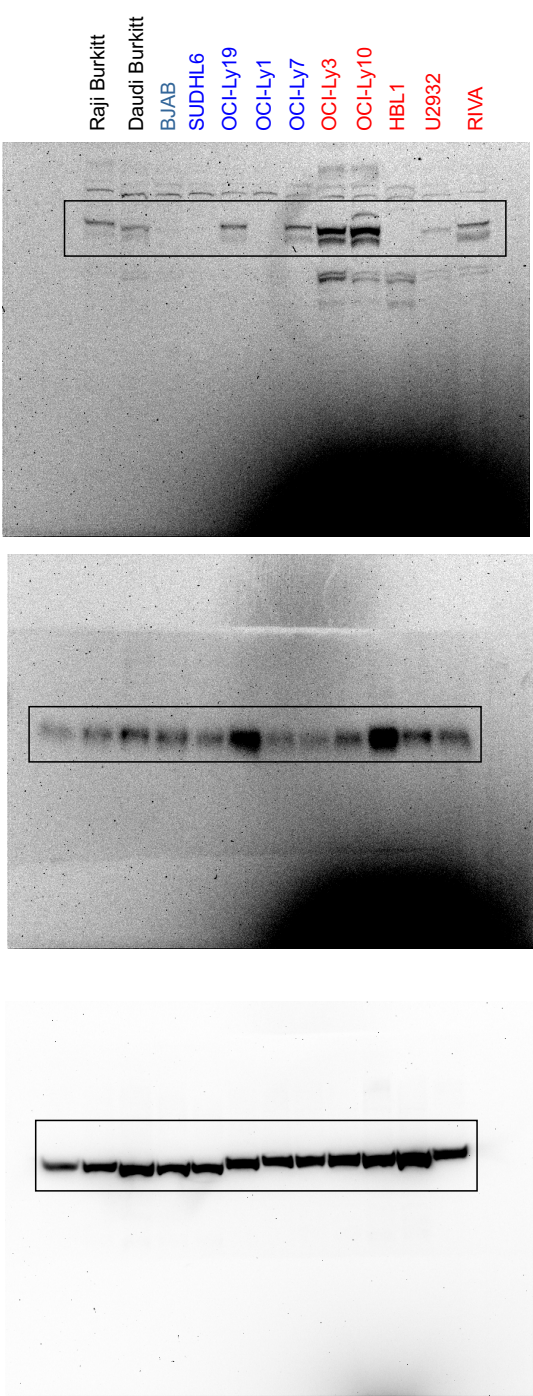

Supplement: Supplementary file 1 [file cancers-14-00338-s001.zip › cancers-1508138-supplementary/cancers-1508138 wb figure.pdf]
